# The Effect of Forage-to-Concentrate Ratio on *Schizochytrium* spp.-Supplemented Goats: Modifying Rumen Microbiota

**DOI:** 10.3390/ani11092746

**Published:** 2021-09-20

**Authors:** Alexandros Mavrommatis, Dimitrios Skliros, Kyriaki Sotirakoglou, Emmanouil Flemetakis, Eleni Tsiplakou

**Affiliations:** 1 Laboratory of Nutritional Physiology and Feeding, Department of Animal Science, School of Animal Biosciences, Agricultural University of Athens, Iera Odos 75, GR-11855 Athens, Greece; mavrommatis@aua.gr; 2 Laboratory of Molecular Biology, Department of Biotechnology, School of Food, Biotechnology and Development, Agricultural University of Athens, Iera Odos 75, GR-11855 Athens, Greece; dsklhros@gmail.com (D.S.); mflem@aua.gr (E.F.); 3 Laboratory of Mathematics and Statistics, Department of Natural Resources and Agricultural Engineering, School of Environment and Agricultural Engineering Agricultural University of Athens, Greece, Iera Odos 75, GR-11855 Athens, Greece; sotirakoglou@aua.gr

**Keywords:** goat, rumen, microbiome, 16s rRNA, qPCR, microalgae, amylase, cellulase, ion-torrent

## Abstract

**Simple Summary:**

The in-depth understanding of rumen functions would be the greatest achievement of animal nutritionists. Hence, plenty of feed additives and various nutritional techniques are studied in modifying and understand the rumen habitat. In our study, we investigated the effect of alteration of the forage: concentrate (F:C) ratio in goats supplemented with the microalgae *Schizochytrium* spp. on rumen microbiota communities and enzymatic activity. Our results suggested that even though specific microbes’ abundance was altered, their corresponding enzymatic potential did not follow the same trend. Nonetheless, principal ruminal functions such as ammonia accumulation, fibrolytic activity, and degradation rate of specific fatty acids were also modified due to dietary intervention.

**Abstract:**

The inclusion of feed additives and the implementation of various nutritional strategies are studied to modify the rumen microbiome and consequently its function. Nevertheless, rumen enzymatic activity and its intermediate products are not always matched with the microbiome structure. To further elucidate such differences a two-phase trial using twenty-two dairy goats was carried out. During the first phase, both groups (20HF n = 11; high forage and 20HG n = 11; high grain) were supplemented with 20 g *Schizochytrium* spp./goat/day. The 20HF group consumed a diet with a forage:concentrate (F:C) ratio of 60:40 and the 20HG-diet consisted of a F:C = 40:60. In the second phase, the supplementation level of *Schizochytrium* spp. was increased to 40 g/day/goat while the F:C ratio between the two groups were remained identical (40HF n = 11; high forage and 40HG n = 11; high grain). By utilizing a next-generation sequencing technology, we monitored that the high microalgae inclusion level and foremost in combination with a high grains diet increased the unmapped bacteria within the rumen. Bacteroidetes and *Prevotella brevis* were increased in the 40HG -fed goats as observed by using a qPCR platform. Additionally, methanogens and Methanomassiliicoccales were increased in high microalgae-fed goats, while *Methanobrevibacter* and Methanobacteriales were decreased. Fibrolytic bacteria were decreased in high microalgae-fed goats, while cellulolytic activity was increased. Ammonia was decreased in high grains-fed goats, while docosapentaenoic and docosahexaenoic acids showed a lower degradation rate in the rumen of high forage-fed goats. The alteration of the F:C ratio in goats supplemented with *Schizochytrium* spp. levels modified both ruminal microbiota and enzymatic activity. However, there was no significant consistency in the relations between them.

## 1. Introduction

The manipulation of the outstanding nature of the rumen microbiome in transforming fibrous and non-fibrous plant materials into valuable nutrients, shaping the future of the dairy sector against modern challenges [1,2]. Amongst these challenges, the production of functional dairy foods, the mitigation of ruminants’ environmental impact, and the improvement of feed efficiency constitute a triptych which attracts the interest of both scientists and industry [3]. With an aim to address the aforementioned issues, several feed additives rich in bioactive compounds have been studied [4]. Microalgae rich in polyunsaturated fatty acids (PUFA) appears to be a sustainable, biotechnological approach to address these issues [5,6]. More specifically, the inclusion of *Schizochytrium* spp. in goats diet fed with a moderate forage:concentrate (F:C) ratio of 50:50 increased milk docosapentaenoic acid (DPA), docosahexaenoic acid (DHA), conjugated linoleic acid (CLA) content and ω3/ω6 ratio [7], while simultaneously decreasing the relative abundance of rumen methanogenic microbes [8,9]. Marine origin PUFA which are included in microalgae biomass could exert toxic effects either against specific rumen microbes [10,11], or bacterial biofilms [12], inhibiting the biohydrogenation process, resulting in a higher proportion of PUFA in milk and meat of ruminants. Further to the disturbance of the rumen biohydrogenation owing to marine PUFAs, their toxicity could also affect both the viability and metabolism of methanogenic microbes [13]. However, extended PUFA overload within the rumen could adversely influence the fermentation balance through alterations in fibrolytic activity. Indicatively, in our previous work, the inclusion of 20 g and 40 g *Schizochytrium* spp./day in goats diet suppressed the abundance of *Ruminococcus flavefaciens* adhered to feed particles [9] while only the high supplementation level (40 g/day) decreased the abundance of species that floated in rumen liquid [8]. 

Furthermore, the F:C ratio could regulate ruminal pH, volatile fatty acids (VFA), NH3-N, and rumen microbial flora as well [14]. An optimal F:C ratio can provide balanced nutrition for ruminants, improved feed conversions and animal performances, and optimized rumen microflora [14]. Notably, the high concentrate ratio in cows’ rumen fluid decreased the abundance of *Fibrobacter succinogenes* affecting the metabolism of cellulose degradation and VFA production [14]. Shifting from low- to high- concentrate diet in cows supplemented with sunflower oil resulted in lower apparent NDF digestibility [15]. Interestingly, linseed oil supplementation tended to increase the ruminal NDF digestibility when combined with a high forage diet [16]. In contrast, the combination of linseed oil with a high concentrate diet negatively affected NDF digestibility [16]. Considering the above evidence, we speculated that by altering the F:C ratio in goats fed with supplementation levels of *Schizochytrium* spp. we could further modify the structure of the rumen microbiome and consequently its biochemistry potential and the production of intermediates. 

Although the advent of meta-omics techniques such as 16 S rRNA sequencing provides a much broader genomic and functional perspective in rumen microbial ecology, which was unfeasible until recently, it is still less cost-effective and more time-consuming compared to a well-designed and accurate qPCR platform. Moreover, the presence of various inhibitors in rumen samples [17] could corrupt downstream processes such as library preparation and ultimately confound the analysis. Due to that, the obtained reads may be hard to annotatd at the species level limiting the given information. Last but not least, the application of a hybrid next-generation sequencing (NGS) platform to explore both prokaryotic and eukaryotic taxa simultaneously further intensifies the complexity and the analysis expenses. Hence, the combination of an initial NGS screening followed by a reliable qPCR platform on the rumen microorganism could provide a broader and dependable approach with the minimum expenses. 

Taking into account the aforementioned facts, this study aimed to evaluate the impact of dietary forage to concentrate ratio (60:40 vs. 40:60) in goats supplemented with *Schizochytrium* spp. levels (20 g vs. 40 g) on rumen microflora communities and key-fermentation activities (amylolysis, proteolysis, and fibrolysis).

## 2. Materials and Methods

### 2.1. Diets and Experimental Design

This study continued the analytical approach initiated in previous research works [18]. The study was conducted with respect to the guidelines of the European Union Directive on the defense of animals used for scientific purposes (EU 63/2010; Council of the European Union 2010). Twenty-two crossbred dairy goats [Alpine × Local (Greek) breeds] at early lactation (70 ± 10 days in milk), were separated into two homogenous groups (n = 11 per group) according to their age (3 to 4 years old), body weight (BW; 50.6 ± 6.1 kg), and (4 fat corrected%) milk yield (FCM_4%_). The experimental trial was divided into two phases (two dietary groups each), which lasted 8 weeks each, with the first 2 weeks being an adaptation period. During the first phase, each goat of both groups (20HF; high forage and 20HG; high grain) was supplemented with 20 g *Schizochytrium* spp./day. The F:C ratio of the 20HF group was 60% forages (alfalfa hay and wheat straw) and 40% concentrate while that of 20HG was 40% forages (alfalfa hay and wheat straw) and 60% concentrates (Table 1). In the second phase, the supplementation level of *Schizochytrium* spp. was increased to 40 g/day/goat while the F:C ratio between the two groups were remained identical (40HF; high forage and 40HG; high grain) (Table 1). *Schizochytrium* spp. is a commercial product traded as DHAgold by the DSM feed industry (DSM Nutritional Products, Marousi, Greece). The *Schizochytrium* spp. were added into concentrate mix aiming to provide 20 and 40 g/goat/day in both high forage (1 Kg concentrate/goat/day; 20 g/*Schizochytrium* spp./Kg in 20HF and 40 g/*Schizochytrium* spp./Kg in 40HF) and high grain (1.3 Kg concentrate/goat/day; 15.4 g/*Schizochytrium* spp./Kg in 20HF and 30.7 g/*Schizochytrium* spp./Kg in 40HF) diets (Table 1 and Table S1). The rations were designed to be isonitrogenous with comparable caloric content based on the National Research Council (2007) values (Table 1). The alfalfa hay, wheat straw and concentrates samples were analyzed for organic matter (OM; Official Method 7.009), dry matter (DM; Official Method 7.007), and crude protein (CP; Official Method 7.016) according to the Association of Official Analytical Chemists (1984) using a Kjeldahl Distillation System (FOSS Kjeltec 8400, Hillerød, Denmark). Neutral detergent fiber (NDF) and acid detergent fiber (ADF) expressed exclusive of residual ash according to the method of Van Soest using an ANKOM 2000 Fiber Analyzer (ANKOM, Macedon, NY, USA) as described by Tsiplakou et al. [19] (Table S2). Non-fibrous carbohydrates were calculated based on the equation described by Cannas et al., [20]. Feed samples were also analyzed for fatty acids profile according to the method of O’Fallon et al., [21] (Table 2). The forages (alfalfa hay and wheat straw) were provided separately from the concentrates. Animals were fed on a group basis, considering their average energy and nutritional requirements in order for the experimental design to represent the typical commercial farm feeding management and the results having practical implications for small ruminants. The available feeding space was higher than the one recommended for adult housed goats (0.33 m per animal) considering to favor simultaneous access and lower competitive interactions at the feeder among animals. Forage was provided with the concentrate in two equal portions after milking. Diet consumption was being recorded on daily basis.

### 2.2. Sample Collection

Rumen samples (n = 88) were collected as previously described by Mavrommatis et al. [9]. More specifically, on the 21st and 42nd experimental day, rumen fluid was gathered from each goat using a stomach tube (flexible PVC tube of 1.5 mm thickness and 10 mm I.D.) and an electric vacuum pump (MZ2CNT, Vacuubrand Gmbh & Co Kg, Wertheim, Germany) before the morning feeding. The stomach tube, when inserted into the cranial dorsal (atrium) as opposed to the central rumen, may affect ruminal fermentation parameters due to the presence of saliva in this site [22]. To reduce saliva contamination and increase the representativeness of ruminal fluid collected from the goats, the stomach tube was inserted to a depth of approximately 120–150 cm, and then the first 20 mL of ruminal fluid was discarded [23]. Immediately after collection, the pH of rumen content was determined using a digital pH meter (pH 210 and HI1236 electrode, Hanna Instruments, Woonsocket, RI, USA) and then the samples were filtered through cheesecloth layers to separate the solid particles, and the rumen liquid (approximately 50 mL) were frozen at −80 °C until the microbial community and fatty acids analyses.

### 2.3. Fatty Acid Determination

Rumen fluid samples were analyzed for fatty acids composition according to the method of O’ Fallon et al. [21]. For the determination of the FA profile, an Agilent 6890 N gas chromatograph equipped with an HP-88 capillary column (60 m × 0.25 mm i.d. with 0.20 µm film thickness, Agilent Technologies, Inc., Santa Clara, CA, USA) and a flame ionization detector (FID) was used. The FID temperature was set at 260 °C, and the chromatographic analysis involved a temperature-programmed run starting at 120 °C and held for 1 min. Then, the ramp was followed by two steps: one step of 1.25 °C/min to 230 °C and another step of 10 °C/min to 240 °C and held for 3 min. Hydrogen was used as the carrier gas with a linear velocity set at 30 cm/s, and helium was the make-up gas. Each peak was identified and quantified using a 37 component FAME mix standard (Supelco, Sigma-Aldrich Co., St. Louis, MO, USA). Additionally, extra standards were used for C_18:2 cis-9, trans-11_, C_18:2 trans-10, cis-12_, and C_18:1 trans-11_ (Supelco, Sigma-Aldrich Co., St. Louis, MO, USA). C_18:1 trans-10_ were identified according to the elution sequence reported by Ratnayake [24] and Shingfield et al. [25]. Tridecanoic acid (C13:0) was used as an internal standard for chromatographic analysis (Fluka, Sigma-Aldrich Co.).

### 2.4. DNA Extraction

DNA extraction was performed as previously described by Mavrommatis et al. [9]. Briefly, DNA extraction was performed using a modified typical cetyltrimethylammonium bromide (CTAB) extraction protocol [26]. Specifically, approximately 1 g of frozen rumen liquid was ground to a fine powder using a mortar and pestle with liquid nitrogen. This powder was then transferred into a preheated lysis buffer (2% CTAB, 100 mM Tris-HCl pH 8.0, 20 mM EDTA, 1.4 M NaCl, 2-mercaptoethanol, and 10 mg/mL proteinase K) and incubated at 57 °C for 2 h. RNase A (10 mg/mL) was added to the sample, followed by incubation for 1 h at 37 °C to remove RNA contamination. Next, we performed a three-fold extraction with an equal volume of chloroform: isoamyl alcohol 24:1 at 4 °C before precipitation with isopropanol overnight at −20 °C. After centrifugation at 7500 g for 15 min at 4 °C, the supernatant was discarded, followed by two washes with 70% and 100% ethanol, respectively. Then, the DNA pellet was resuspended in ultrapure water and purified through a NucleoSpin^®^ Tissue spin column (Macherey-Nagel GmbH & Co., KG, Düren, Germany) according to the manufacturer’s protocol. The quality of the extracted DNA was estimated based on the abundance of DNA content and the levels of impurities in the 260/230 and 260/280 ratios using an ND-1000 spectrophotometer (NanoDrop, Wilmington, DE, USA). Then samples were verified in a 0.70% agarose gel (Figure S1). For the Ion-Torrent sequencing bacteriome screening, the obtained DNA samples were pooled [using the same DNA quantity (50 ng/uL) of each individual sample] per dietary treatment (11 goats × 2 sampling time = 22 samples/treatment) forming four representative samples (n = 1), while for the investigation of the relative abundance of selected microbes the 88 DNA samples were analyzed individually.

### 2.5. Ion Torrent NGS Analysis

After extensive quality assessment with a 2100 Bioanalyzer (Agilent Technologies) and Nanodrop (Thermo Fisher Scientific, Waltham, MA, USA) ruminal bacterial community was studied by NGS using an Ion Torrent Personal Genome Machine (PGM) system (Thermo Fisher Scientifc, Leicestershire, UK) [27]. Seven of the nine hypervariable regions (V2, V3, V4, V6–7, V8, and V9) in the bacterial 16S rRNA gene were targeted. To increase the resolving power of 16S rRNA profiling, primers were designed to amplify variable regions 2, 4, and 8 in a single tube with the resulting amplicon fragments of ~250 base pairs (bp), ~288 bp, and ~295 bp, respectively. In a second single tube, a multiplex PCR reaction targets variable regions 3, 6–7, and 9 with resulting amplicon fragments of ~215 bp, ~260 bp, and ~209 bp, respectively. The primer pools were designed to target >80% of sequences found in the Greengenes database with 100% identity for a primer pair amplifying at least one variable region. The PCR was conducted in duplicate based on manufacturer conditions (Thermo Fisher Scientific).

The PCR amplification products were used to create a library via the Ion Plus Fragment Library Kit (Cat. No. 4471252) with sample indexing using the Ion Xpress™ Barcode Adapters 1–96 Kit (Cat. No. 4474517). Template preparation was performed using the Ion OneTouch™ 2 System and the Ion PGM™ Template OT2 400 Kit (Cat. No. 4479878). Sequencing was conducted using the Ion PGM™ Sequencing 400 Kit (Cat. No. 4482002) on the Ion PGM™ System using the Ion 316™ Chip (Cat. No. 4483324). Primary data analysis was performed with Torrent Suite™ Software v4.0 with automated secondary analysis using Ion Reporter™ Software v4.0.

The 16S rRNA workflow module in Ion Reporter™ Software was classified individual reads using a hybrid of both (i) Basic Local Alignment Search Tool (BLAST) alignment to the curated Greengenes database, which contains >400,000 records that were curated for content and (ii) BLAST alignment to the premium curated MicroSEQ^®^ ID database, a high-quality library of full-length 16S rRNA sequences for >15,000 organisms that have been manually curated for sequence quality, length, annotation, and phylogeny, with frequent taxonomical updates. In the first step, reads are aligned to the MicroSEQ^®^ ID library with any unaligned reads subject to a second alignment to the Greengenes database to achieve rapid and exhaustive bacterial identification.

**Figure 1:**
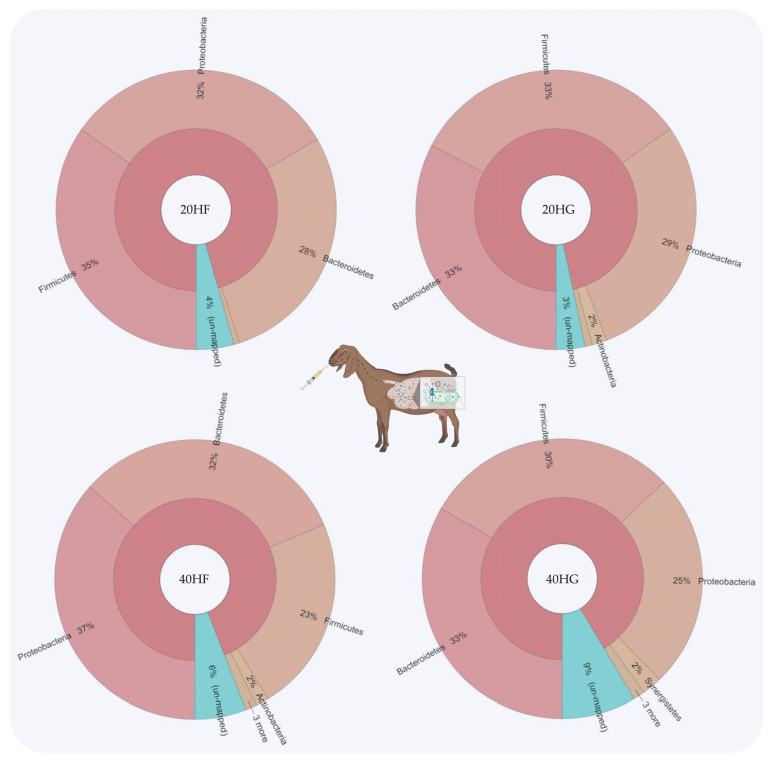
Relative abundance of the most abundant phyla in rumen fluid of goats fed diets (20HF, 20HG, 40HF, and 40HG) with different levels of *Schizochytrium* spp. (20 g and 40 g/goat/day) and two different forage to concentrate ratios (60:40 and 40:60) throughout the experimental period (21 and 42 experimental days) illustrated using Krona software. Additional statistics are available in Table S3.

**Figure 2:**
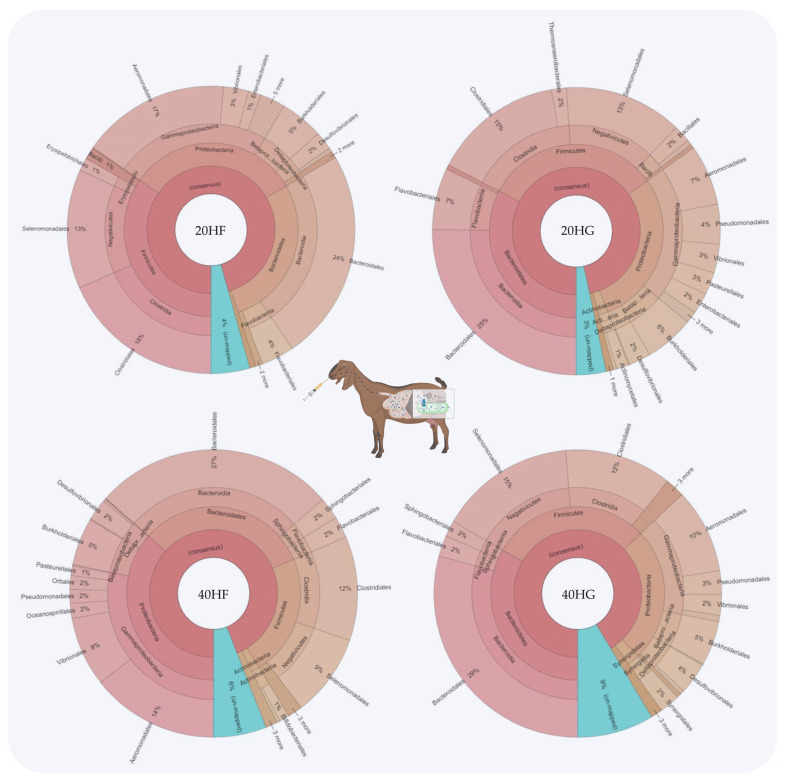
Relative abundance of the most abundant orders in the rumen fluid of goats fed diets (20HF, 20HG, 40HF, and 40HG) with different levels of *Schizochytrium* spp. (20 g and 40 g/goat/day) and two different forage to concentrate ratios (60:40 and 40:60) throughout the experimental period (21 and 42 experimental days) illustrated using Krona software. Additional statistics are available in Table S3.

Taxonomical assignments were reported as a consensus of the results from all of the primers and by each primer, with the option to report multiple taxonomical assignments (slash call). Slash calls can result for a particular variable region when a sequence identifies multiple taxa within a set percentage range. By default, alignment at various taxonomical levels follows the Clinical and Laboratory Standards Institute (CLSI) guidelines requiring the family level to have <97% identity, with genus >97% identity and species >99% identity (Figure 1 and Figure 2). Alpha diversity which describes the diversity within a single sample at the species, genus, and family levels were calculated using the QIIME open-source bioinformatics pipeline.

### 2.6. Relative Abundance of Selected Rumen Microbes

The primer set used for the qPCR, the genomic region of PCR amplification, the PCR efficiency (%), and the slope of the standard curves are presented in Table 3. The primers for amplifying the total bacterial 16S rRNA sequences have been modified to adjust the hybridization temperature to 60 °C [28]. The general anaerobic fungi primers have been designed from multiple alignments of fungal 18S ribosomal and ITS1 gene sequences, which encompassed all available anaerobic fungal sequences [28]. A set of ciliate protozoal PCR primers were designed based on all ruminal protozoan 18S rDNA sequences according to Sylvester et al. [29]. The archaea 16S rRNA gene was amplified using an already published primer pair [30], while for methanogens detection the methyl coenzyme-M reductase subunit A (MCRA) gene was targeted for amplification. This enzyme complex is considered to be unique to, and ubiquitous in, methanogens making it a suitable tool for their exclusive detection [31]. The primer sets for the detection and enumeration of the other bacteria and methanogens populations amplify the 16S rRNA gene sequences, described by Denman and McSweeney [28], Kim et al. [30], Yang et al., [32], Vargas-Bello-Pérez et al., [33], Duval et al., [34], and Elolimy et al. [35] (Table 3). Primers were then associated with sequences accessible at the NCBI via a BLAST search to find out primer specificity. 

Conventional PCR for the validation of the specificity of the chosen primers against target genes was performed in 25 μL reactions with the addition of 2.5 mM MgCl_2_ and employing Taq polymerase following below conditions: initial step at 95 °C for 4 min, and 28 cycles of 95 °C for 30 s, 60 °C for 20 s (or relevant based on primers Tm) and 68 °C for 1 min for elongation. PCR products were analyzed for a unique band and the absence of primer-dimer by running on 2% agarose gels.

**Figure 3:**
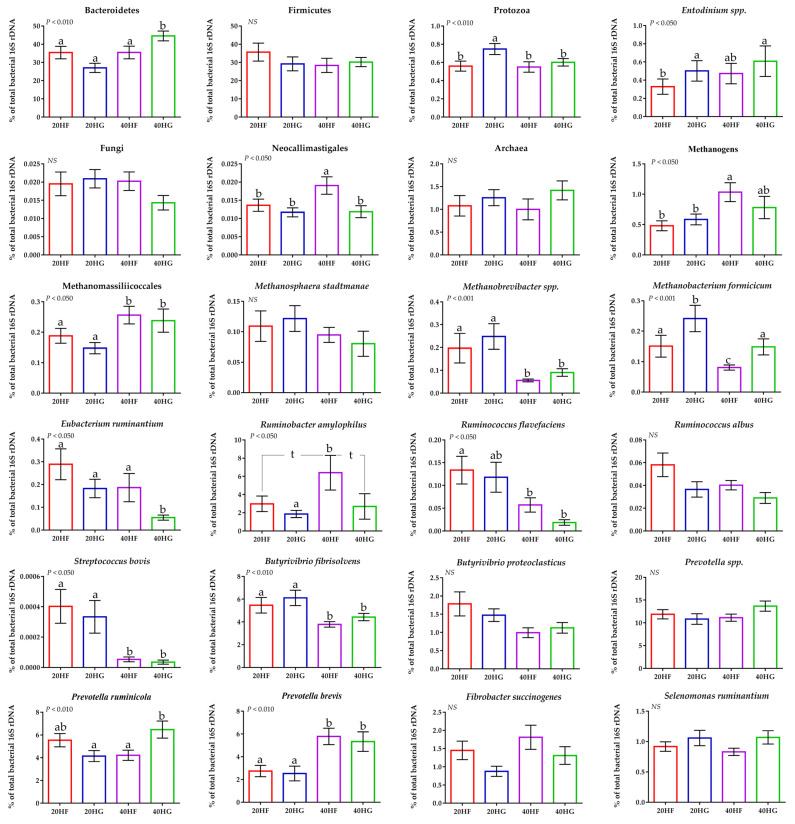
Average changes of target microorganisms in the rumen liquid of goats fed diets (20HF, 20HG, 40HF, and 40HG) with different levels of *Schizochytrium* spp. (20 g and 40 g/goat/day) and two different forage to concentrate ratios (60:40 and 40:60) throughout the experimental period (21 and 42 experimental days) illustrated in column bars (±Standard Error of Means) as a proportion of total rumen bacterial 16S rDNA. Bars with different superscript (a–c) between dietary treatments differ significantly (*p* ≤ 0.05) while t is referred to *p*-value < 0.10, according to the Analysis of Variance (ANOVA) using a general linear model for repeated measures and Post hoc analysis was performed when appropriate using Tukey’s multiple range test. Additional statistics investigating the interaction of microalgae level, forage to concentrate ratio, and the sampling time effect are available in Tables S4 and S5.

Due to the variation of PCR inhibitors in rumen samples, it was vital to validate the PCR efficiency to confirm an accurate quantitation [17]. Consecutive dilutions of pool microbial DNA were amplified (duplicate) by qPCR based on the conditions emerged by primers Tm and product length. Quantitative PCR reaction efficiencies (e) were calculated for the primers presented in Table 3 from a linear regression of the threshold cycle (Ct) for each dilution against the log dilution using the formula: e = 10–1/slope [36]. Primers’ efficiencies varied from 95% to 99%. The relative abundance of microbial populations was expressed as a proportion of total bacterial 16S ribosomal DNA as described by Carberry et al. [17] according to the equation: relative abundance = e (target) − ^(Ct target microorganism-Ct of bacterial 16s rDNA)^, (latest cycle detected = 33,5/40). Changes in the relative abundance of specific microbes were expressed as% of total bacterial 16s rDNA as described by Chen et al. [37] and Carberry et al. [17] (Figure 3). The relative abundance expression of the results is a suitable and accurate method provided that no different taxa are compared, whilst the comparisons are limited to between treatments and time [28]. Quantitative PCRs were performed using a Step-One Plus Real-Time PCR System (Applied Biosystems, Foster City, CA, USA) in a reaction volume of 10 μL: 5 μL SYBRTM Select Master Mix (Thermo Fisher Scientific), 4 μL primers (0.2 μmol each), and 1 μL of DNA (40 ng/uL) as a template according to master mix manufacturer protocol. Primer specificity and formation of primer dimers were explored by dissociation curve analysis (melt curve).

### 2.7. Rumen Enzymes and Ammonia Concentration

During rumen sample collection, 10 mL of rumen digesta were filtered through four layers of cheesecloth and then centrifuged at 13,000× *g* at 4 °C for 5 min. The supernatant was stored in aliquots at −80 °C until the analysis. Each sample was defrosted only once to ensure enzyme functionality. The ammonia-nitrogen (NH_3_-N) concentration, a-amylase, and protease activity were measured using a UV/Vis spectrophotometer (GENESYS 180, Thermo Fisher Scientific). The NH_3_-N concentration was determined using a commercial BUN kit (BIOSIS, Athens, Greece) with proper calibrations using consecutive dilutions (10–100 mg/L) of a 20% ammonia solution (Thermo Fisher Scientific) [38]. Alpha-amylase was assayed by monitoring the reduction of 3,5-dinitrosalicylic acid by released groups from starch at 540 nm according to the method of Worthington Biochemical Corporation (Lakewood, NJ, USA). Protease activity was determined according to the method of Baintner [39]. Proteases split off colored azopeptides from azocasein. The residual azocasein, bacteria, etc. were precipitated with trichloroacetic acid (TCA) and the red color of the azopeptides is then developed with alkali and measured at 440 nm. Cellulase activity was tried to evaluate using the well-described colorimetric method of Azo-CM-Cellulose. However, it was impossible to generate reliable results. Hence, the petri dish method described by Abe et al. [40] was used (Figure S2). Briefly, a medium containing 37 mM KH_2_PO_4_; 11 mM K_2_HPO_4_; 0.4 mM MgSO_4_·7H_2_O; 7.6 mM (NH_4_)_2_SO_4_; and 27 mM microcrystalline cellulose at pH 5.5; and 15 g/L agar (w/v) was used. After the inoculum of rumen fluid, the dishes were incubated at 50 °C, for 16 h before evaluation. After this, 5 mL of the iodine solution was spread to visualize the hydrolytic halo. The same procedure was followed for xylanase activity based on the method of Kalim and Ali [41] with some modifications. Briefly, the cellulase’s medium was used by substituting the microcrystalline cellulose with 10 g/L xylan from corn core, while the incubation took place at 37 °C, for 20 h. Standard curves were obtained by consecutive dilutions of endo-cellulase (A. niger) and endo-1–4-beta-Xylanase M1 (T. viride) respectively (Megazyme, Wicklow, Ireland). ImageJ densitometry software (Version 1.6, National Institute of Health, Bethesda, MD, USA) was used for clearance zone quantitative analysis [42].

**Table 3:** Sequences of primers used for qPCRs, genomic regions of PCR amplification, primer efficiency, standard curve slope, amplicon size and hybridization temperature.

Target Species	Genomic Region of PCR Amplification	Primer Sequencing	Primer Efficiency%	Slope	Amplicon bp	Τm °C	References
Total bacteria	16s rRNA	F: 5’-CGGCAACGAGCGCAACCC-3’	98	−3.378	130	60	[28]
		R: 5’-CCATTGTAGCACGTGTGTAGCC-3’					
Bacteroidetes	16s rRNA	F: 5’-GGARCATGTGGTTTAATTCGATGAT-3’	98	−3.36	126	62	[43]
		R: 5’-AGCTGACGACAACCATGCAG-3’					
Firmicutes	16s rRNA	F: 5’-GGAGYATGTGGTTTAATTCGAAGCA-3’	97	−3.39	126	62	[43]
		R: 5’-AGCTGACGACAACCATGCAC-3’					
Archaea	16s rRNA	F: 5’-GAGGAAGGAGTGGACGACGGTA-3’	96	−3.43	233	60	[44]
		R: 5’-ACGGGCGGTGTGTGCAAG-3’					
Protozoa	18s rRNA	F: 5’-GCTTTCGWTGGTAGTGTATT-3’	97	−3.39	223	55	[30]
		R: 5’-CTTGCCCTCYAATCGTWCT-3’					
*Entodinium*	18s rRNA	F: 5’-GAGCTAATACATGCTAAGGC-3’	97	−3.39	317	59	[30]
		R: 5’-CCCTCACTACAATCGAGATTTAAGG-3’					
Total fungi	18s rRNA ITS1	F: 5’-GAGGAAGTAAAAGTCGTAACAAGGTTTC-3’	98	−3.38	120	58	[28]
		R: 5’-CAAATTCACAAAGGGTAGGATGATT-3’					
Neocallimastigales	18s rRNA ITS1	F: 5’-TTGACAATGGATCTCTTGGTTCTC-3’	96	−3.43	110	63	[30]
		R: 5’-GTGCAATATGCGTTCGAAGATT-3’					
Methanogen	mcrA	F: 5’-TTCGGTGGATCDCARAGRGC-3’	95	−3.44	140	58	[30]
		R: 5’-GBARGTCGWAWCCGTAGAATCC-3’					
Methanomassiliicoccales	16s rRNA	F: 5′-TTCTGGGGTAGGGGTAAAATC-3′	97	−3.40	149	62	[30]
		R: 5′-GTCTGCAGCGTTTACACCCT-3′					
Methanobacteriales	16s rRNA	F: 5’- CGWAGGGAAGCTGTTAAGT-3’	95	−3.45	343	55	[30]
		R: 5’-TACCGTCGTCCACTCCTT-3’					
*Methanobrevibacter* spp.	16s rRNA	F: 5’-TGGGAATTGCTGGWGATACTRTT-3’	95	−3.46	231	60	[30]
		R: 5’-GGAGCRGCTCAAAGCCA-3’					
*Methanosphaera stadtmanae*	16s rRNA	F: 5’-CTTAACTATAAGAATTGCTGGAG-3’	97	−3.39	150	58	[30]
		R:5’-TTCGTTACTCACCGTCAAGATC-3’					
*Butyrivibrio fibrisolvens*	16s rRNA	F: 5’-TAACATGAGAGTTTGATCCTGGCTC-3’	97	−3.39	136	58	[32]
		R: 5’-CGTTACTCACCCGTCCGC-3’					
*Butyrivibrio proteoclasticus*	16s rRNA	F: 5’-TCCGGTGGTATGAGATGGGC-3’	98	−3.38	185	60	[33]
		R: 5’-GTCGCTGCATCAGAGTTTCCT-3’					
*Eubacterium ruminantium*	16s rRNA	F: 5’-CTCCCGAGACTGAGGAAGCTTG-3’	98	−3.36	184	62	[35]
		R: 5’-GTCCATCTCACACCACCGGA-3’					
*Ruminococcus flavefaciens*	16s rRNA	F: 5’-CGAACGGAGATAATTTGAGTTTACTTAGG-3’	95	−3.42	132	60	[28]
		R: 5’-CGGTCTCTGTATGTTATGAGGTATTACC-3’					
*Fibrobacter succinogenes*	16s rRNA	F: 5’-GCGGGATTGAATGTACCTTGAGA-3’	98	−3.39	204	60	[32]
		R: 5’-TCCGCCTGCCCCTGAACTATC-3’					
*Ruminococcus albus*	16s rRNA	F: 5’-CCCTAAAAGCAGTCTTAGTTCG-3’	98	−3.38	175	62	[30]
		R: 5’-CCTCCTTGCGGTTAGAACA-3’					
*Ruminobacter amylophilus*	16s rRNA	F: 5’-ATGCAAGTCGAACGGTAACAGCAGG-3’	96	−3.42	115	65	[34]
		R: 5’-GCACCCGTTTCCAGGTGTTGTCC-3’					
*Streptococcus bovis*	16s rRNA	F: 5’-TTCCTAGAGATAGGAAGTTTCTTCGG-3’	96	−3.43	127	57	[35]
		R: 5’-ATGATGGCAACTAACAATAGGGGT-3’					
*Selenomonas ruminantium*	16s rRNA	F: 5’-CAATAAGCATTCCGCCTGGG-3’	99	−3.35	138	57	[35]
		R: 5’-TTCACTCAATGTCAAGCCCTGG-3’					
*Prevotella* sp.	16s rRNA	F: 5’-GGTTCTGAGAGGAAGGTCCCC-3’	96	−3.42	121	60	[30]
		R: 5’-TCCTGCACGCTACTTGGCTG-3’					
*Prevotella brevis*	16s rRNA	F: 5’-GGTTTCCTTGAGTGTATTCGACGTC-3’	98	−3.38	219	64	[30]
		R:5’-CTTTCGCTTGGCCGCTG-3’					
*Prevotella ruminicola*	16s rRNA	F: 5’-GAAAGTCGGATTAATGCTCTATGTTG-3’	97	−3.39	74	63	[30]
		R:5’-CATCCTATAGCGGTAAACCTTTGG-3’					

### 2.8. Statistics

Dataset was evaluated in SPSS.IBM software (v 20.0) and the results are depicted as mean ± standard error of means (SEM). The effect of dietary treatment between the four groups was assessed by performing a GLMM for repeated measures analysis of variance. The dietary treatments (D) (D = 20HF, 20HG, 40HF, and 40HG) were defined as the fixed factor and the sampling time (S) as the repeated measure, while their interactions (*D* × *S*) were also assessed, according to the following model:

(1) $$Y_{ijkl}=\mu +D_{i}+S_{j}+A_{k}+{\left(D\times S\right)}_{ij}+e_{ijkl}$$

where *Υijkl* is the dependent variable, *μ* the overall mean, *Di* the effect of dietary treatment (*I* = 4; 20HF, 20HG, 40HF, and 40HG), *S_j_* the effect of sampling time (*j* = 2; 21st and 42nd experimental day), *A_k_* the animal’s random effect, (*D × S*)*ij* the interaction between dietary treatments and sampling time, and *e_ijkl_* the residual error. A total of 88 observations (11 goats × 4 dietary groups × 2 sampling times) emerged for each variable. Post-hoc analysis was applied when appropriate using Tukey’s multiple range test. For all tests, the significance level was set at *p* = 0.05. To simplify the visualization of these results, interleaved bars were depicted using GraphPad Prism 6.0 (2012, GraphPad Software Inc., San Diego, CA, USA). 

Ruminal fatty acid profile, the abundance of selected rumen microorganisms by qPCR, and the enzymatic activities were also analyzed using a GLMM for three-way repeated-measures ANOVA, considering the forage to concentrate ratio (F/C) (60/40, 40/60) as the between-subjects factor and microalgae level (A) (20 g, 40 g) and sampling time (S) (21st, 42nd experimental day) as within-subjects’ factors and the interactions among them according to the model:

(2) $$Y_{ijklm}=\mu +{\left(F/C\right)}_{i}+A_{j}+S_{k}+G_{l}+{\left(F/C\times A\right)}_{ij}+{\left(F/C\times S\right)}_{ik}+{\left(A\times S\right)}_{jk}+{\left(F/C\times A\times S\right)}_{ijk}+e_{ijklm}$$

where $Y_{ijklm}$ is the dependent variable, μ the overall mean, ${\left(F/C\right)}_{i}$ the effect of forage to concentrate ratio (*i* = 2; 60/40 and 40/60), $A_{j}$ the effect of microalgae level (*j* = 2; 20 g and 40 g), $S_{k}$ (*k =* 2; 21st and 42nd experimental day), $G_{l}$ the animal’s random effect, ${\left(F/C\times A\right)}_{ij},\;{\left(F/C\times S\right)}_{ik},\;{\left(A\times S\right)}_{jk},\;{\left(F/C\times A\times S\right)}_{ijk}$ the two-way and three-way interactions between the aforementioned factors of the experiment and $e_{ijklm}$ the residual errors. Posthoc analysis was applied when appropriate using Tukey’s multiple range test. For all tests, the significance level was set at *p* = 0.05.

Discriminant analyses were also performed (variables were entered independent together) on rumen fluid fatty acids and the abundance of selected rumen microorganisms by qPCR pooled data to establish those variables capable of distinguishing and classifying samples amongst the four dietary groups (20HF, 20HG, 40HF, and 40HG). Wilk’s lambda (λ) criterion was used for assessing discriminant functions [45]. Twenty and twenty-four variables for rumen fluid fatty acid profile and the abundance of selected rumen microorganisms by qPCR were entered to create two models to distinguish the eighty-eight samples of each case (4 groups × 11 goats/group × 2 sampling time). 

## 3. Results

### 3.1. Feed Intake 

The mean wheat straw intake was decreased by 34% and 50% in the 20HF and 40HF groups, respectively (Table 4). The mean concentrate intake was also decreased in both 40HF and 40HG groups by 16%. These changes also decrease the microalgae intake since they have been supplemented into the concentrates (40HF; 33.7 g and 40HG; 33.2 g vs. the planned of 40 g; Table 4). However, the planned F:C ratios and NDF to starch proportion were not considerably modified (Table 4).

### 3.2. Sequencing and Quality Filtering

A total of 412,838 reads were generated from a total of four samples, with a mean of 103,209 reads per sample. After quality filtering, 29,796 (13.8%) high-quality sequences were successfully mapped (Table 5). Rarefaction curves of the bacterial population at the genera taxonomic level show that all samples reached a plateau phase, meaning that an increase in the number of sequences will not impact the number of genera detected (Figure S3). Primer sets coverage is presented in Supplementary Materials (Figure S4).

### 3.3. Diversity, Richness, and Screening of the Ruminal Bacteriome Using Ion-Torrent

Shannon Wiener and Simpson values in genus level showed a slightly higher diversity in high concentrate diets (20HG and 40HG), compared to those that were fed with high forage levels (20HF and 40HF). However, in family level which portraying a more dependable depth of analysis, no difference existed (Table 6). 

Bacteroidetes were increased in the 20HG, 40HF, and 40HG groups compared to the 20HF group, while Firmicutes showed a lower abundance in the 40HF rumen fluid (Figure 1). Proteobacteria proportions were increased in the 40HF group compared to other dietary treatments (Figure 1). Interestingly, the high microalgae level in the goats’ diet (40HF and 40HG) caused an increase in the un-mapped species, while in the case of high microalgae level combined with high grain diet the proportion of un-mapped bacteria reach 9% (Figure 1). In order level, the proportion of *Flavobacteriales* was increased in 20HG-fed goats while *Vibrionales* showed a peak in the 40HF group (Figure 2). *Selenomanadales* were decreased in the 40HF-fed goats compared to other groups (Figure 2). *Bacteroidales* and *Desulfovibrionales* were increased in the 40HG-fed goats while *Clostridiales* were increased in the 20HF group (Figure 2). Further metagenomic information at the family level and visualizations using Krona software are available in the Supplementary Materials (Table S3 and Figure S5).

### 3.4. Relative Abundance of Selected Microorganism in the Rumen Fluid Using qPCR Platform

Using a qPCR-specific platform, the Bacteroidetes were increased significantly (*p* < 0.010) in the 40HG compared to 20HF, 20HG, and 40HF-fed goats, while Firmicutes were not considerably altered (Figure 3; Table S4). Protozoa abundance was increased in the rumen liquid of 20HG-fed goats (*p* < 0.010) while the dominant ciliate protozoa; *Entodinium* spp. were decreased (*p* < 0.050) in the 20HF goats compared to other dietary treatments (Figure 3; Table S4). Furthermore, protozoa showed a significant upsurge (*p* < 0.050) in the high grains-fed goats rumen liquid (Table S5). The single order of ruminal anaerobic fungi; Neocallimastigales, were increased (*p* < 0.050) in the 40HF-fed goats (Figure 3; Table S4), while tended to decrease (*p* = 0.099) in response to high grain diets and to increase (*p* = 0.084) with the upsurge of microalgae supplementation (Table S5). Total methanogenic archaea were increased in the high microalgae diets and significantly (*p* < 0.050) in the high forage group (40HF) (Figure 3; Tables S4 and S5). Methanomassiliicoccales (also known as rumen cluster C) were significantly (*p* < 0.001) increased in high microalgae-fed goats (40HF and 40HG), while the abundance of *Methanobrevibacter* spp. was significantly (*p* < 0.001) decreased in high microalgae- compared to the low microalgae fed goats (Table S5). The relative abundance of Methanobacteriales order was significantly (*p* < 0.001) increased in the rumen fluid of 20HG-fed goats, while in 40HG considerable mitigation was observed compared to 20HF and 40HG treatments (Figure 3; Table S4). The relative abundance of *Eubacterium ruminantium* was significantly decreased (*p* < 0.050) in 40HG-fed goats (Figure 3; Table S4). *Ruminococcus flavefaciens* was significantly decreased in high microalgae-fed goats (40HF and 40HG), while *Ruminococcus albus* were not altered (Figure 3; Tables S4 and S5). The relative abundance of *Ruminobacter amylophilus* was significantly (*p* < 0.050) increased in 40HF- compared to 20HG-fed goats, while the 20HF and 40HG showed a tendency (*p* < 0.100) to decreased compared to 40HF-diet (Figure 3; Table S4). *Streptococcus bovis* and *Butyrivibrio fibrisolvens* were significantly decreased (*p* < 0.010) in high microalgae-fed goats (40HF and 40HG) (Table S5). Even though the relative abundance of *Prevotella* spp was not affected, the proportion of *Prevotella ruminicola* was increased in 40HG- compared to the 20HG and 40HF-fed goats (Figure 3; Table S4). On the other hand, *Prevotella brevis* were significantly (*p* < 0.010) increased in high microalgae-fed goats (40HF and 40HG) (Figure 3; Tables S4 and S5).

20HF (22 DNA samples pooled: 11 goats × 2 sampling time): 20 g *Schizochytrium* spp. and high forage diet (60:40); 20HG (22 DNA samples pooled: 11 goats × 2 sampling time): 20 g *Schizochytrium* spp. and high grain diet (40:60); 40HF (22 DNA samples pooled: 11 goats × 2 sampling time): 40 g *Schizochytrium* spp. and high forage diet (60:40); 40HG (22 DNA samples pooled: 11 goats × 2 sampling time): 40 g *Schizochytrium* spp. and high grain diet (40:60).

20HF (22 DNA samples pooled: 11 goats × 2 sampling time): 20 g *Schizochytrium* spp. and high forage diet (60:40); 20HG (22 DNA samples pooled: 11 goats × 2 sampling time): 20 g *Schizochytrium* spp. and high grain diet (40:60); 40HF (22 DNA samples pooled: 11 goats × 2 sampling time): 40 g *Schizochytrium* spp. and high forage diet (60:40); 40HG (22 DNA samples pooled: 11 goats × 2 sampling time): 40 g *Schizochytrium* spp. and high grain diet (40:60).20HF (n = 11 goats): 20 g *Schizochytrium* spp. and high forage diet (60:40); 20HG (n = 11 goats): 20 g *Schizochytrium* spp. and high grain diet (40:60); 40HF (n = 11 goats): 40 g *Schizochytrium* spp. and high forage diet (60:40); 40HG (n = 11 goats): 40 g *Schizochytrium* spp. and high grain diet (40:60).

### 3.5. Ruminal Fatty Acid Profile

Caproic (C_6:0_) and capric (C_10:0_) acid levels were significantly increased (*p* < 0.001 and *p* < 0.010 respectively) in 40HF and 40HG-fed goats (Table 7). Lauric acid (C_12:0_) was increased (*p* < 0.050) in the 40HG-fed goats (Table 7). Myristic acid (C_14:0_) was significantly increased (*p* < 0.001) in 40HG-fed goats, while in the 20HG-fed goats a significant reduction was observed compared to the 20HF and 40HF-fed goats (Table 7). Pentadecanoic acid (C_15:0_) was significantly decreased (*p* < 0.050) in the high grain- compared to the high forage-fed goats (Table S6). Palmitic (C_16:0_) and palmitoleic acid (C_16:1_) were significantly increased (*p* < 0.010 and *p* < 0.050 respectively) in the rumen of high microalgae-fed goats (Table S6). Stearic acid (C_18:0_) was decreased (*p* < 0.001) in the rumen of high microalgae-fed goats (Table S6). Vaccenic acid (C_18:1 trans 11_) was significantly increased (*p* < 0.050) in the rumen of high grain-fed goats, while a tendency to increase (*p* = 0.080) was found in high microalgae fed groups (Table S6). Linoleic acid (C_18:2 n6 cis_) was significantly decreased (*p* < 0.001) in the rumen of high microalgae-fed goats, while a tendency to decrease (*p* = 0.083) was found in high grain-fed groups (Table S6). Besides, the combination of high microalgae inclusion level and high grain (40HG) diet further decreased (*p* < 0.001) the proportion of linoleic acid in rumen fluid (Table 7). Conjugated C_18:2_ isomers were increased (*p* < 0.010) in the rumen fluid of the 20HF-fed goats (Table S6). Linolenic acid (C_18:3 n3 cis_) was significantly decreased (*p* < 0.010) in the rumen of high grain-fed goats, while a tendency to increase (*p* = 0.078) was found in high microalgae fed goats (Table S6). Both docosapentaenoic acid (C_22:5 n-6_; DPA), docosahexaenoic acid (C_22:6 n-3_; DHA) were significantly decreased (*p* < 0.001) in the rumen of high grain-fed goats, while were significantly increased (*p* < 0.001) in high microalgae fed groups (Table S6).

### 3.6. Discriminant Analysis of Rumen Fatty Acid and Selected Microorganism Abundance

**Figure 4:**
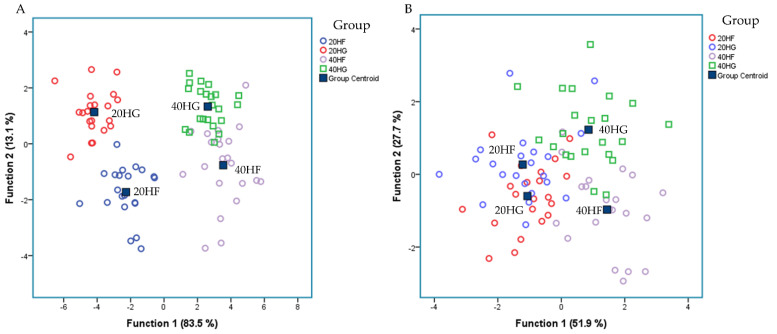
Discriminant plots separating (**A**) the four dietary treatments (20HF; blue, 20HG; green, 40HF; red, and 40HG; pink) according to pooled data of two sampling time (21st and 42nd experimental day) on the rumen fluid fatty acid profile and (**B**) the four dietary treatments (20HF; blue, 20HG; green, 40HF; red, and 40HG; pink) according to pooled data of two sampling time (21st and 42nd experimental day) on the selective ruminal microorganism by qPCR.

Figure 4A depicts a discriminant plot of the ruminal fatty acid profile of the four dietary treatments (20HF; blue, 20HG; red, 40HF; pink, and 40HG; green) throughout the experimental period. The proportions of the samples that were correctly classified were 94.2%. Wilks’ lambda was observed at 0.021 for Function (1) (*p* < 0.001) and 0.254 for Function (2) (*p* < 0.001), while the proportions of C_22:2 n6_, C_18:2 n6 cis_, C_18:3 n3_, C_6:0_, C_14:0_, C_20:0_, C_16:0_, and C_22:6 n3_ were the variables that contributed the most based on a step wise method. The four dietary treatments are clearly classified apart without observing any overlapping in the observations. However, within Function (1) which describes 83.5% of the model, the level of microalgae possesses the dominant role. 

Figure 4B depicts the second discriminant plot of the relative abundance of selected microorganisms quantified by qPCR of the four dietary treatments (20HF; blue, 20HG; red, 40HF; pink, and 40HG; green) throughout the experimental period. The proportions of the samples that were correctly classified were 82.6%. Wilks’ lambda was observed at 0.1511 for Function (1) (*p* < 0.001) and 0.365 for Function (2) (*p* = 0.005), while the proportions of *R. Flavefaciens, Prevotella brevis*, *B. Fibrisolvens*, *Prevotella ruminocola*, and *Neocallimastigales* were the variables that contributed the most based on a step wise method. The centroids of the four dietary treatments are classified apart. However, a few overlapping were observed amongst the observations. 

20HF (n = 11 goats): 20 g *Schizochytrium* spp. and high forage diet (60:40); 20HG (n = 11 goats): 20 g *Schizochytrium* spp. and high grain diet (40:60); 40HF (n = 11 goats): 40 g *Schizochytrium* spp. and high forage diet (60:40); 40HG (n = 11 goats): 40 g *Schizochytrium* spp. and high grain diet (40:60).

### 3.7. Enzyme Activities, Ammonia Concentration, and Ruminal PH

**Figure 5:**
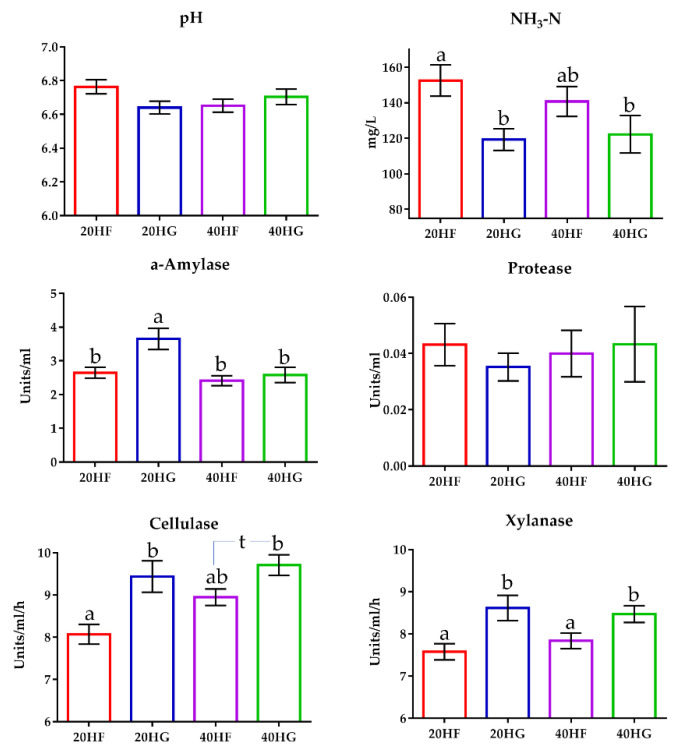
The mean ruminal pH, ammonia concentration, a-amylase, protease, cellulase, and xylanase activity of goats fed diets (20HF, 20HG, 40HF, and 40HG) with different levels of microalgae Schizochytrium spp (20 g and 40 g/day/goat) and two different forage to concentrate ratios (60:40 and 40:60) throughout the experimental period (21st and 42nd experimental day) illustrated in bar graphs ± SEM. Bars with different superscript (a, b) between dietary treatments differ significantly (*p* ≤ 0.05) according to the Analysis of Variance (ANOVA) using a general linear model for repeated measures and Post hoc analysis was performed when appropriate using Tukey’s multiple range test. Additional statistics investigating the interaction of microalgae level, forage to concentrate ratio, and the sampling time effect are available in Tables S7 and S8. 20HF (n = 11 goats): 20 g Schizochytrium spp. and high forage diet (60:40); 20HG (n = 11 goats): 20 g Schizochytrium spp. and high grain diet (40:60); 40HF (n = 11 goats): 40 g Schizochytrium spp. and high forage diet (60:40); 40HG (n = 11 goats): 40 g Schizochytrium spp. and high grain diet (40:60).

Ammonia concentration was significantly decreased (*p* < 0.05) in the rumen fluid of the high grains-fed goats (Figure 5; Table S7). In detail, ammonia was determined by 24% lower in the rumen fluid of the 20HG- compared to the 20HF-fed goats (*p* < 0.05) (Table S8). Still, ammonia was also decreased by 14% in the rumen fluid of the 40HG- compared to the 40 H-fed goats, however, the results did not significantly differ in this case (Table S8). Ruminal pH did not differ significantly (*p* > 0.05) amongst the dietary treatments (Tables S7 and S8). The α-amylase activity tended to increase (*p* = 0.081) in high grains-fed goats, while was significantly decreased in the high microalgae-fed goats (Figure 5; Table S8). Additionally, α-amylase activity was increased (*p* < 0.01) in the 20HG group (Figure 5; Table S8). Cellulase and xylanase activities were determined by measuring the clearance zones of rumen inoculum in cellulose and xylan agar Petri dishes, respectively. Cellulase activity was increased both in high grains- (*p* < 0.05) and high microalgae-fed goats (*p* < 0.05) (Figure 5; Table S7). More specifically, cellulase activity was increased (*p* < 0.01) by 16% in the rumen fluid of the 20HG- compared to the 20HF-fed goats (Figure 5; Table S8). Similarly, an increase of 9.5% was observed in the rumen fluid of the 40HG- compared to the 40HF-fed goats (*p* < 0.10) (Figure 5). Xylanase activity was also increased (*p* < 0.05) in the high grains-fed goats (Figure 5; Table S7). In detail, xylanase activity was significantly increased (*p* < 0.05) in the rumen fluid of 20HG and 40HG groups by 14% and 8% respectively, compared to 40HF and 40HG (Figure 5; Table S8).

## 4. Discussion

Although the microalgae levels stalwartly modified goats’ rumen microbiome, alterations in dietary forage to concentrate ratio unveiled important new insights as well. More specifically, the effect of microalgae level per se was quite predictable considering our preliminary studies on both goats’ rumen liquid and solid fractions under comparable experimental conditions [8,9]. However, their interaction with the F:C ratio appeared to robustly orchestrate rumen habitat as well. Some discrepancies in the abundance levels of dominant phyla between NGS and qPCR approaches may lie in both different analytical workflows (pool DNA samples on NGS vs. individually on qPCR) and the dissimilar amplification region within the 16s rRNA. Although these observations were not subversive, this partial incompatibility between the two approaches remains a debatable issue requiring more research. 

### 4.1. Combined Microalgae Level and F:C Ratio Modulated Microbiome Structure

The richness and diversity of ruminal bacteriome are important indicators of normal rumen biochemistry processes. Metagenomics indicated that shifting from high grain to high forage diet tended to a slightly less diverse rumen bacteriome and a minor habitat filtering of species as obtained by Shannon and Simpson indices only in genus level. Rumen diversity is usually decreased in high concentrate diets attributed to pH reduction [46]. In our study, aiming to design trials with practical implementation, the F:C ratios ranged within ordinary conditions. Hence, the ruminal pH was not considerably altered. Shifting cows’ diet from 70% forage to 30% increased rumen microbial diversity based on the Simpson index as well [47].

Bacteroidetes, Firmicutes, and Proteobacteria appear to constitute the core bacteriome in goats’ rumen amongst the dietary treatments. Interestingly, the inclusion of 40 g *Schizochytrium* spp. in goats’ diet and foremost in combination with high grain ratio radically increased the proportion of unmapped species even though the annotation has been performed using a hybrid model of two databases. The exploration of unmapped bacteria may unlock a deeper understanding of rumen habitat willing to bridge the gap between microbiome structure and rumen biochemistry.

Bacteroidetes were increased in high microalgae-fed goats due to the increment of some *Prevotella* species (e.g., *P. ruminicola* and *P. brevis*) as was observed through the qPCR approach, the *Prevotellaceae* family was increased in high microalgae-fed goats (40HF and 40HG) showed a peak in 40HG goats (Table S3). The in vitro inclusion of PUFA rich oil mixes (sunflower, fish oil, algae oil) increased the Bacteroidetes *Prevotella* spp. in sheep rumen fluid as well [48]. The *Prevotellaceae* family may have been able to expand their niche as a result of the general reduction in numbers of other species majorly belonging to Firmicutes phylum. However, considering that the rumen microbiome-assembly process is rather driven by niche modification and not niche preemption, such changes in inhabitation require deeper research attention [49].

The sum of dominant phyla Bacteroidetes and Firmicutes showed minimum abundance in the 20HG-fed goats, while protozoa abundance was found significantly higher in this treatment. Besides, protozoa relative abundance was also increased in the high grain-fed goats, while the dominant protozoal ciliates genus; Entodinium, were only numerically increased. Shifting ruminants’ diet from forage to high concentrates increased the abundance of protozoa species with the most intense that of Entodinium [50]. Mao et al. [51] and Hook et al. [52] reported that Entodinium species are linearly engulfed starch particles with the upsurge of concentrates within the rumen. This ability may alter the degradation rate of starch in the rumen and consequently its digestibility and propionate production [53]. Considering that recent studies demonstrate that unsaturated fatty acids such as these presented in microalgae biomass could prevent the formation of bacterial biofilm returning them into a planktonic lifestyle [12], while ciliate protozoa appear to be the major planktonic bacterial predators [54], it is plausible to assume that the bacteria abundance may be suppressed in the 20HG-fed goats due to protozoa grazing properties. 

Overlooking the mutualistic dependence of protozoa with the methanogens and their synergistic impact on methane production [55], their increase in our study may conceal an important aspect regarding the enrichment of dairy products with PUFA. More specifically, protozoa facilitate the escape of unsaturated fatty acids from the rumen by engulfing plant chloroplasts resulting in their release into the duodenum [56]. It should be mentioned here, that despite the high accumulation of PUFA in *Schizochytrium* spp. biomass, these heterotrophic species lack of chloroplasts [57]. Instead, the de novo FA synthesis has been evolutionarily shifted onto an ectoplasmic network [58]. Nevertheless, it remains an open question whether the protozoal species could effectively protect microalgae’ PUFAs using an analogous mechanism. Furthermore, considering that up to 75% of microbial fatty acids present in the rumen may be of protozoal origin, and that protozoal biomass tends to accumulate high proportions of certain biologically important molecules which are produced as intermediates of the incomplete biohydrogenation (CLA and VA) [59], further set of evidence arising to support a balancing perspective of protozoa against the unsaturated FA hydrogenating rumen habitat [60].

Even though total anaerobic fungi were remained unaffected under the dietary alterations, the dominant order of Neocallimastigales was increased in the 40HF-fed goats. Fungi abundance and diversity is usually decreased in the rumen of goats switched from low to high-grain diet [61]. Furthermore, in our previous experimental trial, the inclusion of 40 g *Schizochytrium* spp. in goats diet tended to increase the relative abundance of anaerobic fungi in rumen liquid compared to those consumed 20 g [9]. Taking into account the aforementioned, the increase of Neocallimastigales may lie in the synergetic effect of high forage level and microalgae inclusion in the 40HF-fed goats. 

Archaea did not differ among the treatment while methanogens on *MCRA* and Methanomassiliicoccales (also known as rumen cluster C) on *16 s* were increased in high microalgae-fed goats. In contrast, the relative abundance of *Methanobrevibacter* and Methanomicrobiales were declined in high microalgae-fed goats. Even though the results regarding the Euryarchaeota diversity and abundance are quite controversial, it has been proposed that the composition rather than the size of the methanogen community in the rumen is more closely associated with CH_4_ production [5]. For example, in both cattle and sheep, no differences were noted in the overall relative abundance of Archaea between high and low CH_4_-emitting animals [62,63,64]. Nevertheless, the abundance of *Methanobrevibacter*, a predominant member of the rumen archaeal community, is firmly associated with methane production. These fluctuations are attributed to the different expression levels of the various forms of methyl-coenzyme M reductase within the archaeal cells [5]. To conclude, there is a challenging gap between methanogens communities and methane production in rumen under targeted nutritional strategies that need to be further explored. 

### 4.2. PUFA and F:C Ratio as Biohydrogenation Regulators

A high accumulation of vaccenic acid in the rumen liquid of high concentrate-fed goat was observed, while the microalgae level did not considerably affect its proportion. Interestingly, the aforementioned pattern was not completely followed in the next biohydrogenation step (stearic acid formation). More specifically, the F:C ratio did not affect the proportion of ruminal stearic acid compared to the microalgae level which did, indicating changes in *Butyrivibrio proteoclasticus* abundance [65,66]. Indeed, *B. proteoclasticus* was decreased in the rumen liquid of high microalgae- compared to low microalgae-fed goats. This positive correlation signifying an orchestration mechanism of PUFA towards *B. proteoclasticus* activity. However, the accumulation of biohydrogenation intermediates is not inextricably linked with the involved *Butyrivibrio* species [67,68,69,70] since (i) their DNA footprint is not associated with their metabolic activity [71] and ii) there is recent evidence regarding the synergetic action of different bacterial species involved in biohydrogenation process. Such species are belonging to the *Lachnospiraceae* family and more specifically to *Blautia* genus [67]. 

Considering the decline of ruminal linoleic acid and the tendency for higher accumulation of vaccenic acid in the high microalgae-fed goats it could be speculated that the ruminal lipolysis and the first step of biohydrogenation are promoted by the higher fat level. The degradation of linoleic acid under the inclusion of *Schizochytrium* spp. in goats diet portrays a consistent trend since it has been revealed previously as well [9].

Although, the high NDF levels stimulate the rumen biohydrogenation [72], interestingly both ruminal DPA and DHA concentrations were reported higher in high forage-fed goats. Τhe mechanism underlies the higher degradation of these long-chain PUFA in high grain diets remains unclear. However, a negative correlation of DPA and DHA proportions and *B. fibrisolvens* relative abundance was observed. This correlation may involve *B. fibrisolvens* in these long-chain PUFAs degradation. The rumen biotransformation of DPA and DHA is still in its infancy, however, our set of evidence showing that the high forage diets may result in a higher transfer rate is quite crucial for their on-farm implementation since their average apparent transfer efficiency is rather low [73].

### 4.3. Rumen Biochemistry: Microbes Abundance vs. Metabolic Implications

Although the predominant cellulolytic bacteria (*E. ruminantium*,* R. flavefaciens*, and *R. albus*) [74] in treated groups were progressively decreased compared to 20HF-fed goats with the most intense decline revealed in the 40HG-fed, the enzymatic cellulolytic activity was significantly increased in high grain-fed goats subverts the linkage between microbes’ abundance and nutrients degradation rate. Both *Ruminococcus* and *Eubacterium* abundance are negatively affected by high grain proportion in ruminants’ diets [75] and by the inclusion of polyunsaturated fatty acids as has been previously reported in vitro [11] and in vivo [8]. Investigating the inhibition of cellulolytic bacteria in presence of PUFA, there is strong evidence involving severe toxicity of PUFA on the cell membrane particularly of Gram-positive bacteria resulting in metabolic imbalances [10,11]. Additionally, recent evidence proposes that PUFA toxicity does not lie in bacteria disruption per se rather in the prevention of the biofilm formation in Gram-positive bacteria [12]. In contrast, the mechanism underlies the F:C on ruminal bacteriome appears to be more well-studied as lie in changes in pH [76]. However, in our study, the ruminal pH was not considerably affected indicating that any further decline in cellulolytic bacteria in high-grain fed goats may be attributed to the synergistic action of F:C and PUFA inclusion. 

There is still a high interest in the unbalanced nature of the upsurge in the cellulolytic activity while the predominant bacteria involved were not portrayed such a trend. Our hypothesis is based on the higher availability of energy through the increase of starch in the high-grain fed goats. More specifically, due to the potentially high metabolic fermentation of starch in the high-grain fed goats which was partially confirmed by the higher activity of alpha-amylase in the 20HG diet, the available energy in bacteria may be utilized to form glycogen and other carbohydrate compounds in a process named “reserve carbohydrate synthesis” which is activated during energy excess within the rumen [77]. Thus, it could be assumed that fibrolysis was expanded by the cellulolytic microbes in response to the high availability of accessible energy even though their population tended to decrease. The progressive replacement of starch with soluble NDF in the rumen using the rumen simulation technique (RUSITEC) significantly decreased the cellulase activity in the liquid fraction as well [78]. Notably, ruminal supplementation with high available energy through molasse also improved the activities of fibrolytic enzymes in sheep [79]. This set of evidence supports the idea that the enzymatic activity of rumen is not strictly linked with their corresponding bacterial DNA footprint. 

Another hypothesis that may explain the inconsistency between fibrolytic abundance and enzymatic activity in high grain-fed goats might lie in hydrogen production within the rumen. More specifically, based on our preliminary studies indicating that *Schizochytrium* spp. inclusion tended to decrease the methanogenic archaea abundance [8,9], while in in vitro studies the methane formation was significantly mitigated [80], it could be assumed that the ability of rumen to neutralize the formed hydrogen produced by fibrolytic microbes was suppressed as well. Additionally, the high forage diets promote the growth of fibrolytic bacteria and consequently the formation of hydrogen. Taking into account the aforementioned, we speculated that in high forage diets (20HF and 40HF) an extended amount of H_2_ was accumulated since was unable to be neutralized by methanogens. The extended amount of H_2_ may inhibit the normal functioning of microbial enzymes involved in electron transfer reactions, particularly that of NADH dehydrogenase, resulting in NADH accumulation and ultimately reduce rumen fibrolytic activity [81].

The α-amylase activity was increased in the 20HG-fed goats. Interestingly, in the aforementioned dietary group, there was observed a significant increase in the abundance of protozoa. As previously stated, the high availability of starch may stimulate the engulfing of starch particles in protozoan cells changing its degradation within the rumen. Still, discrepancies between rumen microbial communities and enzymatic activity were observed as well. More specifically, the dominant amylolytic bacteria; *Ruminobacter amylophilus* was significantly increased in the 40HF-fed goats, while a multi-factorial analysis further showing that the relative abundance of *R. amylophilus* was higher in high microalgae-fed goats (40 g) and tended to increase in high forage diets. The NGS analysis further confirmed the aforementioned tendency, showing a higher abundance of *Ruminobacter* genus in the high forage-fed goats. Additionally, our preliminary work revealed a tendency for higher *R. amylophilus* abundance in both liquid [9] and solid [8] fraction of goats fed with 40 compared to 20 g *Schizochytrium* spp./day. However, *Streptococcus bovis* is also considered to be an important bacterium with high amylolytic potential within the rumen [82]. The radical decrease of the relative abundance of *S. bovis* in high microalgae-fed goats may counteract the opposite trend which was observed for *R. amylophilus.* These imbalances in microbes’ abundance in combination with differences in their metabolism due to dietary treatments entangling the explanation regarding the observed ruminal amylolytic activity.

Regarding the rumen proteolytic activity, even though both *Prevotella ruminicola* and *P. brevis* were increased in the 40HG- and high microalgae-fed goats respectively, the overall proteolytic activity was not affected. However, ammonia concentration was significantly decreased in the rumen of goats fed with high grain diets. Considering that ruminal ammonia was exceeded the limit of 50 mg/L, the microbial synthesis was only energy-depended [83]. Thus, it is plausible to assume that the decline of ammonia in high grain-fed goats is related to the high energy availability due to starch content matching the requirements for microbial synthesis. The lower ammonia concentration indicates an improved nitrogen utilization which may positively develop both the overall economic sustainability and environmental impact.

## 5. Conclusions

The combination of microalgae *Schizochytrium* spp. either at 20 or 40 g/day with high forage diet appears to be an ideal formula to improve the transfer efficiency of both DPA and DHA. However, shifting from high forage to a high grain diet improved the fibrolytic activity and ammonia utilization and therefore may increase microbial protein synthesis. Nevertheless, further research should be conducted under a metabolic spectrum (transcriptomic and proteomic approaches) to validate the aforementioned observations. NGS using the Ion-Torrent technology appears to be a valuable tool for predicting and orchestrating nutrient utilization, achieved through targeted dietary interventions. However, its applications should be limited to genus or even better to family level and accompanied by further validation approaches. The biochemistry of the rumen microbiome depicts an abyssal chapter which understanding shaping the future of the ruminants’ sector.

## Figures and Tables

**Table 1:** Ration components (Kg/goat/day) and chemical composition (g/day) of the diets were administered to the four groups (20HF, 20HG, 40HF, and 40HG) of goats involved in the trials.

	Treatment			
	20HF	20HG	40HF	40HG
Diet components (Kg per goat)				
Alfalfa hay	1.2	0.7	1.2	0.7
Wheat straw	0.3	0.18	0.3	0.18
Concentrate mix	1	1.3	1	1.3
*Schizochytrium* spp. (g)	20	20	40	40
Forage to Concentrate ratio	1.5:1 (60:40)	0.88:1.3 (40:60)	1.5:1 (60:40)	0.88:1.3 (40:60)
Dry Matter	2282	1989	2298	2000
Ash	188	144	192	142
Crude Protein	312	311	312	311
Ether Extract	82.3	87.9	90.3	97.0
Ash-free NDF treated with amylase	932	712	931	710
Acid Detergent Fiber	608	399	605	409
Non Fibrous Carbohydrate	987	925	976	920
Starch	474	542	462	542
NDF/Starch	2.0	1.3	2.0	1.3
Energy NEL MJ/goat/day	12.5	12.2	12.5	12.2

20HF: 20 g *Schizochytrium* spp. and high forage diet (60:40); 20HG: 20 g *Schizochytrium* spp. and high grain diet (40:60); 40HF: 40 g *Schizochytrium* spp. and high forage diet (60:40); 40HG: 40 g *Schizochytrium* spp. and high grain diet (40:60).

**Table 2:** Alfalfa hay, wheat straw and concentrates fatty acid profile (FA) (% of total FA).

Fatty Acid	Concentrates				Forages	
	20HF	20HG	40HF	40HG	Alfalfa hay	Wheat Straw
Myristic acid (C_14:0_)	2.48	2.12	3.1	3.18	6.2	0
Palmitic acid (C_16:0_)	21.94	22.99	20.22	23.77	36.77	29.88
Stearic acid (C_18:0_)	1.92	2.06	1.55	1.87	2.33	4.86
Oleic acid (C_18:1 cis-9_)	28.95	31.83	22.13	27.08	2.49	34.77
Linoleic acid (C_18:2n−6 cis_)	31.96	31.04	31.09	27.76	18.27	21.95
Eicosanoic acid (C_20:0_)	0.22	0.23	0.17	0.2	0.64	0.82
Linolenic acid (C_18:3n−3_)	1.07	0.96	1.13	0.88	30.68	1.86
Eicosatrienoic acid (C_20:3n3_)	0.44	0.37	0.56	0.47	1.5	1.37
Lignoceric acid (C_24:0_)	0.32	0.25	0.26	0.25	0	0.73
Docosapentaenoic acid (C_22:5n−6_)	2.42	1.92	4.7	3.78	0	0
Docosahexaenoic acid (C_22:6n−3_)	6.71	5.25	13.76	10.21	0	0

20HF: 20 g *Schizochytrium* spp. and high forage diet (60:40); 20HG: 20 g *Schizochytrium* spp. and high grain diet (40:60); 40HF: 40 g *Schizochytrium* spp. and high forage diet (60:40); 40HG: 40 g *Schizochytrium* spp. and high grain diet (40:60).

**Table 4:** Feed intake on a fresh matter basis (Kg/goat and percentage of consumed quantities compared to given) and nutrients consumption (g) of the four groups (20HF, 20HG, 40HF, and 40HG) of goats involved in the trials.

	Treatment			
	20HF	20HG	40HF	40HG
**Diet Consumption kg**				
Alfalfa hay	1.2 (100)	0.7 (100)	1.2 (100)	0.7 (100)
Wheat straw	0.2 (66)	0.18 (99)	0.15 (50)	0.16 (90)
Concentrate mix	0.97 (97)	1.29 (99)	0.84 (84)	1.09 (84)
*Schizochytrium* spp. g	19.3 (97)	19.8 (99)	33.7 (84)	33.2 (83)
Forage to Concentrate ratio	1.4:0.97 (59:41)	0.88:1.29 (40:60)	1.35:0.84 (61:39)	0.76:1.09 (41:59)
**Nutrients Intake**				
Dry Matter	2161	1980	2010	1788
Ash	179	144	173	131
Crude Protein	305	309	286	276
Ether Extract	79	87	76	83
Ash-free NDF amylase treated	853	709	788	649
Acid Detergent Fiber	555	398	515	383
Non Fibrous Carbohydrate	954	920	866	810
Starch	460	538	393	459
NDF/Starch	1.9	1.3	2.0	1.4

20HF (n = 11 goats): 20 g *Schizochytrium* spp. and high forage diet (60:40); 20HG (n = 11 goats): 20 g *Schizochytrium* spp. and high grain diet (40:60); 40HF (n = 11 goats): 40 g *Schizochytrium* spp. and high forage diet (60:40); 40HG (n = 11 goats): 40 g *Schizochytrium* spp. and high grain diet (40:60).

**Table 5:** Sequencing quality and mapped reads per sample.

Sequencing Quality	Treatments			
	20HF	20HG	40HF	40HG
BP cutoff	150	150	150	150
Total number of reads	108,680	96,337	135,739	90,381
Number of valid reads	53,722	47,693	67,877	45,205
Number of reads ignored	46,901 *	40,717 *	56,359 *	38,522 *
Mapped reads in sample	6524	6743	10,835	6109
Un-Mapped reads in sample	297	233	683	574

* (due to low number of copies <10); 20HF (22 DNA samples pooled: 11 goats × 2 sampling time): 20 g *Schizochytrium* spp. and high forage diet (60:40); 20HG (22 DNA samples pooled: 11 goats × 2 sampling time): 20 g *Schizochytrium* spp. and high grain diet (40:60); 40HF (22 DNA samples pooled: 11 goats × 2 sampling time): 40 g *Schizochytrium* spp. and high forage diet (60:40); 40HG (22 DNA samples pooled: 11 goats × 2 sampling time): 40 g *Schizochytrium* spp. and high grain diet (40:60).

**Table 6:** Alpha diversity of ruminal bacteriome in family and genus level.

	Treatments			
	20HF	20HG	40HF	40HG
**Family level**				
Shannon index	4.101	4.317	4.042	3.865
Simpson index	0.911	0.928	0.899	0.880
**Genus level**				
Shannon index	2.513	2.875	2.610	3.018
Simpson index	0.724	0.825	0.747	0.851

20HF (22 DNA samples pooled: 11 goats × 2 sampling time): 20 g *Schizochytrium* spp. and high forage diet (60:40); 20HG (22 DNA samples pooled: 11 goats × 2 sampling time): 20 g *Schizochytrium* spp. and high grain diet (40:60); 40HF (22 DNA samples pooled: 11 goats × 2 sampling time): 40 g *Schizochytrium* spp. and high forage diet (60:40); 40HG (22 DNA samples pooled: 11 goats × 2 sampling time): 40 g *Schizochytrium* spp. and high grain diet (40:60).

**Table 7:** The mean individual fatty acids (FA) (% of total FA) in rumen fluid of goats fed diets (20HF, 20HG, 40HF, and 40HG) with different levels of microalgae *Schizochytrium* spp. (20 g and 40 g/day/goat) and two different forage to concentrate ratios (60:40 and 40:60) throughout the experimental period (21st and 42nd experimental day) using a GLM model for repeated measure.

Fatty Acids	Dietary Treatment (D)					Sampling Time (S)			Effect		
	20HF	20HG	40HF	40HG	SEM	21	42	SEM	D	S	D × S
C_6:0_	0.41 ^a^	0.51 ^a^	1.79 ^b^	1.76 ^b^	0.182	1.29	1.11	0.124	***	NS	**
C_8:0_	1.83	1.42	2.12	1.85	0.209	1.96 ^a^	1.65 ^b^	0.125	NS	*	***
C_10:0_	0.95 ^a^	0.76 ^a^	1.24 ^b^	1.44 ^b^	0.124	1.08	1.12	0.095	**	NS	NS
C_12:0_	0.63 ^a^	0.74 ^a^	0.87 ^a^	1.20 ^b^	0.123	0.95	0.77	0.081	*	NS	NS
C_14:0_	3.90 ^a c^	3.15 ^b^	4.51 ^c^	4.91 ^d^	0.208	4.42 ^a^	3.85 ^b^	0.146	***	**	*
C_14:1_	1.15	1.05	1.10	1.02	0.065	1.14	1.02	0.040	NS	*	NS
C_15:0_	1.10 ^a^	0.89 ^b^	1.12 ^a^	0.94 ^a b^	0.066	1.03	0.99	0.039	*	NS	NS
C_16:0_	28.19	28.23	28.71	29.16	0.286	28.38	28.76	0.197	NS	NS	NS
C_16:1 n−7_	0.50	0.44	0.58	0.72	0.088	0.63 ^a^	0.49 ^b^	0.052	NS	*	NS
C_17:0_	0.66 ^a c^	0.48 ^a^	1.08 ^b^	0.94 ^c b^	0.125	0.86	0.72	0.084	**	NS	NS
C_18:0_	19.77 ^a^	18.75 ^a^	11.47 ^b^	5.66 ^b^	2.690	14.07	13.75	1.475	**	NS	*
C_18:1 *trans*_	1.92	1.86	1.82	2.06	0.207	1.81	2.02	0.123	NS	NS	NS
C_18:1 *trans 11*_	9.59 ^a^	16.26 ^b^	12.66 ^a^	20.23 ^b^	2.366	14.48	14.94	1.258	*	NS	NS
C_18:1 *trans 10*_	1.19	1.47	1.21	1.54	0.165	1.31	1.39	0.098	NS	NS	NS
C_18:1 cis-9_	9.68	10.72	9.42	10.64	0.483	10.09	10.13	0.288	NS	NS	NS
C_18:2 n−6 trans_	0.83	0.76	0.66	0.53	0.089	0.75	0.65	0.057	NS	NS	***
C_18:2 n−6 cis_	4.82 ^a^	4.57 ^a^	3.43 ^b^	2.61 ^c^	0.251	3.84	3.87	0.161	***	NS	NS
C_18:3 n−3_	1.19 ^a^	0.87 ^a^	1.56 ^b^	0.94 ^a^	0.136	1.17	1.11	0.072	**	NS	NS
C_18:2 Conjugated_	1.39 ^a^	1.07 ^b^	1.09 ^b^	0.83 ^b^	0.099	0.99 ^a^	1.20 ^b^	0.066	**	*	NS
C_22:5 n−6_	2.96 ^a^	1.76 ^b^	4.04 ^c^	3.43 ^d a^	0.197	2.97	3.13	0.131	***	NS	*
C_22:6 n−3_	7.26 ^a^	4.07 ^b^	9.49 ^c^	7.44 ^d a^	0.451	6.89	7.26	0.297	***	NS	NS

Means with different superscript letters (a–c) between dietary groups and sampling time differ significantly; * *p* < 0.05, ** *p* < 0.01, *** *p* < 0.001. 20HF (n = 11 goats): 20 g *Schizochytrium* spp. and high forage diet (60:40); 20HG (n = 11 goats): 20 g *Schizochytrium* spp. and high grain diet (40:60); 40HF (n = 11 goats): 40 g *Schizochytrium* spp. and high forage diet (60:40); 40HG (n = 11 goats): 40 g *Schizochytrium* spp. and high grain diet (40:60).

## Data Availability

Data are contained within the article and Supplementary Materials.

## Supplementary Materials

The following are available online at https://www.mdpi.com/article/10.3390/ani11092746/s1, Figure S1: DNA quality of samples used for Ion Torrent, Figure S2: Cellulase and Xylanase enzymatic activity using the petri method, Figure S3: Rarefaction curves and alpha diversity of the four dietary groups (20HF, 20HG, 40HF, and 40HG) at genera taxonomic level, Figure S4: Primers coverage in the four samples of goats rumen, Figure S5: Metagenomic visualization using Krona software are available in family level, Figure S6. The mean individual fatty acids (FA) (% of total FA) in rumen fluid of goats fed diets (20HF, 20HG, 40HF, and 40HG) with different levels of Schizochytrium spp. (20 g and 40 g/goat/day) and two different forage to concentrate ratios (60:40 and 40:60) throughout the experimental period (21 and 42 experimental days) illustrated in column bars (±Standard Error of Means); Table S1: Ingredients of concentrate (g/Kg) of the four diets, Table S2: Feed chemical composition (%), Table S3: Relative abundance of rumen fluid bacteriome of goats fed diets (20HF, 20HG, 40HF and 40HG) with different levels of Schizochytrium spp. (20 g and 40 g/goat/day) and two different forage to concentrate ratios (60:40 and 40:60) throughout the experimental period (21 and 42 experimental days), Table S4: Relative abundance of several microorganisms to the total bacterial in the rumen fluid of goats fed diets (20HF, 20HG, 40HF, and 40HG) with different levels of microalgae Schizochytrium spp (20 g and 40 g/day/goat) and two different forage to concentrate ratios (60:40 and 40:60) throughout the experimental period (21th and 42th experimental day) using a GLM model for repeated measure, Table S5: Relative abundance of several microorganisms to the total bacterial in the rumen fluid of goats fed diets (20HF, 20HG, 40HF, and 40HG) with different levels of microalgae Schizochytrium spp (20 g and 40 g/day/goat) and two different forage to concentrate ratios (60:40 and 40:60) throughout the experimental period (21th and 42th experimental day) using a GLM model for three-way repeated measures, Table S6: The mean individual fatty acids (FA) (% of total FA) in rumen fluid of goats fed diets (20HF, 20HG, 40HF, and 40HG) with different levels of microalgae Schizochytrium spp. (20 g and 40 g/day/goat) and two different forage to concentrate ratios (60:40 and 40:60) throughout the experimental period (21th and 42th experimental day) investigating the three major factors (forage-to-concentrate ratio, algae level, and sampling time), Table S7: The mean rumen pH values, ammonia concentration, a-amylase, protease, cellulase, and xylanase activity of goats fed diets (20HF, 20HG, 40HF, and 40HG) with different levels of microalgae Schizochytrium spp (20 g and 40 g/day/goat) and two different forage to concentrate ratios (60:40 and 40:60) throughout the experimental period (21th and 42th experimental day) using a GLM model for three-way repeated measures, Table S8: The mean rumen pH values, ammonia concentration, a-amylase, protease, cellulase, and xylanase activity of goats fed diets (20HF, 20HG, 40HF, and 40HG) with different levels of microalgae Schizochytrium spp (20 g and 40 g/day/goat) and two different forage to concentrate ratios (60:40 and 40:60) throughout the experimental period (21th and 42th experimental day).

## References

[1] Hassan F.U., Arshad M.A., Ebeid H.M., Rehman M.S., Khan M.S., Shahid S., Yang C. (2020). Phytogenic Additives Can Modulate Rumen Microbiome to Mediate Fermentation Kinetics and Methanogenesis Through Exploiting Diet-Microbe Interaction. Front. Vet. Sci..

[2] Varijakshapanicker P., Mckune S., Miller L., Hendrickx S., Balehegn M., Dahl G.E., Adesogan A.T. (2019). Sustainable livestock systems to improve human health, nutrition, and economic status. Anim. Front..

[3] Mlambo V., Mnisi C.M. (2019). Optimizing ruminant production systems for sustainable intensification, human health, food security and environmental stewardship. Outlook Agric..

[4] Halmemies-Beauchet-Filleau A., Rinne M., Lamminen M., Mapato C., Ampapon T., Wanapat M., Vanhatalo A. (2018). Review: Alternative and novel feeds for ruminants: Nutritive value, product quality and environmental aspects. Animal.

[5] Abbott D.W., Aasen I.M., Beauchemin K.A., Grondahl F., Gruninger R., Hayes M., Huws S., Kenny D.A., Krizsan S.J., Kirwan S.F. (2020). Seaweed and Seaweed Bioactives for Mitigation of Enteric Methane: Challenges and Opportunities. Animals.

[6] Nguyen Q.V., Malau-Aduli B.S., Cavalieri J., Malau-Aduli A.E.O., Nichols P.D. (2019). Enhancing Omega-3 Long-Chain Poly-unsaturated Fatty Acid Content of Dairy-Derived Foods for Human Consumption. Nutrients.

[7] Mavrommatis A., Tsiplakou E. (2020). The impact of the dietary supplementation level with Schizochytrium sp., on milk chemical composition and fatty acid profile of both blood plasma and milk of goats. Small Rum. Res..

[8] Mavrommatis A., Skliros D., Simoni M., Righi F., Flemetakis E., Tsiplakou E. (2021). Alterations in the Rumen Particle-Associated Microbiota of Goats in Response to Dietary Supplementation Levels of *Schizochytrium* spp.. Sustainability.

[9] Mavrommatis A., Sotirakoglou K., Skliros D., Flemetakis E., Tsiplakou E. (2021). Dose and time response of dietary supplementation with Schizochytrium sp. on the abundances of several microorganisms in the rumen liquid of dairy goats. Livest. Sci..

[10] Maia M.R.G., Chaudhary L.C., Figueres L., Wallace R.J. (2007). Metabolism of polyunsaturated fatty acids and their toxicity to the microfora of the rumen. Anton. Leeuw..

[11] Maia M.R., Chaudhary L.C., Bestwick C.S., Richardson A.J., McKain N., Larson T.R., Graham I.A., Wallace R.J. (2010). Toxicity of unsaturated fatty acids to the biohydrogenating ruminal bacterium, Butyrivibrio fibrisolvens. BMC Microbiol..

[12] Yuyama K.T., Rohde M., Molinari G., Stadler M., Abraham W.-R. (2020). Unsaturated Fatty Acids Control Biofilm Formation of Staphylococcus aureus and Other Gram-Positive Bacteria. Antibiotics.

[13] Patra A., Park T., Kim M., Yu Z. (2017). Rumen methanogens and mitigation of methane emission by anti-methanogenic compounds and substances. J. Anim. Sci. Biotechnol..

[14] Wang L., Zhang G., Li Y., Zhang Y. (2020). Effects of High Forage/Concentrate Diet on Volatile Fatty Acid Production and the Microorganisms Involved in VFA Production in Cow Rumen. Animals.

[15] Bayat A.R., Ventto L., Kairenius P., Stefański T., Leskinen H., Tapio I., Negussie E., Vilkki J., Shingfield K.J. (2017). Dietary forage to concentrate ratio and sunflower oil supplement alter rumen fermentation, ruminal methane emissions, and nutrient utilization in lactating cows. Transl. Anim. Sci..

[16] Ueda K., Ferlay A., Chabrot J., Loor J.J., Chilliard Y., Doreau M. (2003). Effect of linseed oil supplementation on ruminal digestion in dairy cows fed diets with different forage:concentrate ratios. J. Dairy Sci..

[17] Carberry C.A., Kenny D.A., Han S., McCabe M.S., Waters S.M. (2012). Effect of Phenotypic Residual Feed Intake and Dietary Forage Content on the Rumen Microbial Community of Beef Cattle. Appl. Environ. Microbiol..

[18] Mavrommatis A., Sotirakoglou K., Kamilaris C., Tsiplakou E. (2021). Effects of Inclusion of Schizochytrium spp. and Forage-to-Concentrate Ratios on Goats’ Milk Quality and Oxidative Status. Foods.

[19] Tsiplakou E., Mavrommatis A., Skliros D., Sotirakoglou K., Flemetakis E., Zervas G. (2018). The effects of dietary supplementation with rumen-protected amino acids on the expression of several genes involved in the immune system of dairy sheep. J. Anim. Physiol. Anim. Nutr..

[20] Cannas A., Tedeschi L.O., Fox D.G., Pell A.N., Van Soest P.J. (2004). A mechanistic model for predicting the nutrient requirements and feed biological values for sheep. J. Anim. Sci..

[21] O’Fallon J.V., Busboom J.R., Nelson M.L., Gaskins C.T. (2007). A direct method for fatty acid methyl ester synthesis: Application to wet meat tissues, oils, and feedstuffs. J. Anim. Sci..

[22] Shen J.S., Chai Z., Song L.J., Liu J.X., Wu Y.M. (2012). Insertion depth of oral stomach tubes may affect the fermentation parameters of ruminal fluid collected in dairy cows. J. Dairy Sci..

[23] Ramos-Morales E., Arco-Pérez A., Martín-García A.I., Yáñez-Ruiz D.R., Frutos P., Hervás G. (2014). Use of stomach tubing as an alternative to rumen cannulation to study ruminal fermentation and microbiota in sheep and goats. Anim. Feed Sci. Technol..

[24] Ratnayake W.M.N., Sébédio J.L., Christie W.W. (2018). Analysis of trans fatty acids. Trans Fatty Acids in Human Nutrition.

[25] Shingfield K.J., Reynolds C.K., Hervas G., Griinari J.M., Grandison A.S., Beever D.E. (2006). Examination of the persistency of milk fatty acid composition responses to fish oil and sunflower oil in the diet of dairy cows. J. Dairy Sci..

[26] Sharma R., Mahla H.R., MohPatra T., Bhargva S.C., Sharma M.M. (2003). Isolating plant genomic DNA without liquid nitrogen. Plant. Mol. Biol. Rep..

[27] de la Fuente G., Belanche A., Girwood S.E., Pinloche E., Wilkinson T., Newbold C.J. (2014). Pros and cons of ion-torrent next generation sequencing versus terminal restriction fragment length polymorphism T-RFLP for studying the rumen bacterial community. PLoS ONE.

[28] Denman S.E., McSweeney C.S. (2006). Development of a real-time PCR assay for monitoring anaerobic fungal and cellulolytic bacterial populations within the rumen. FEMS Microbiol. Ecol..

[29] Sylvester J.T., Karnati S.K., Yu Z., Morrison M., Firkins J.L. (2004). Development of an assay to quantify rumen ciliate protozoal biomass in cows using real-time PCR. J. Nutr..

[30] Kim M., Park T., Yu Z. (2017). Invited Review—Metagenomic investigation of gastrointestinal microbiome in cattle. Asian-Australas. J. Anim. Sci..

[31] Luton P.E., Wayne J.M., Sharp R.J., Riley P.W. (2002). The mcrA gene as an alternative to 16S rRNA in the phylogenetic analysis of methanogen populations in landfill. Microbiology.

[32] Yang S.L., Bu D.P., Wang J.Q., Hu Z.Y., Li D., Wei H.Y., Loor J.J. (2009). Soybean oil and linseed oil supplementation affect profiles of ruminal microorganisms in dairy cows. Animal.

[33] Vargas-Bello-Pérez E., Cancino-Padilla N., Romero J., Garnsworthy P.C. (2016). Quantitative analysis of ruminal bacterial populations involved in lipid metabolism in dairy cows fed different vegetable oils. Animal.

[34] Duval S.M., McEwan N.R., Graham R.C., Wallace R.J., Newbold C.J. (2007). Effect of a blend of essential oil compounds on the colonization of starch-rich substrates by bacteria in the rumen. J. Appl. Microbiol..

[35] Elolimy A.A., Arroyo J.M., Batistel F., Iakiviak M.A., Loor J.J. (2018). Association of residual feed intake with abundance of ruminal bacteria and biopolymer hydrolyzing enzyme activities during the peripartal period and early lactation in Holstein dairy cows. J. Anim. Sci. Biotechnol..

[36] Pfaffl M.W. (2001). A new mathematical model for relative quantification in real-time RT-PCR. Nucleic Acids Res..

[37] Chen X.L., Wang J.K., Wu Y.M., Liu J.X. (2008). Effects of chemical treatments of rice straw on rumen fermentation characteristics, fibrolytic enzyme activities and populations of liquid- and solid-associated ruminal microbes in vitro. Anim. Feed Sci. Tech..

[38] Ikutakazuhiro K., Okadakeiji N., Yasuda O. (2004). Simplified Method Using Rumen Ammonium Nitrogen as a Blood-test Reagent. J. Jpn. Vet. Med. Assoc..

[39] Baintner K. (2010). Determination of Proteolytic Activity of Rumen Liquor with Azocasein. Zent. Für Veterinärme-Dizin Reihe A.

[40] Abe C.A.L., Faria C.B., De Castro F.F., De Souza S.R., Santos F.C.D., Da Silva C.N., Tessmann D.J., Barbosa-Tessmann I.P. (2015). Fungi Isolated from Maize (Zea mays L.) Grains and Production of Associated Enzyme Activities. Int. J. Mol. Sci..

[41] Kalim B., Ali N.M. (2016). Optimization of fermentation media and growth conditions for microbial xylanase production. Biotech.

[42] Schneider C.A., Rasband W.S., Eliceiri K.W. (2012). NIH Image to ImageJ: 25 years of image analysis. Nature Methods.

[43] Bahl M.I., Bergström A., Licht T.R. (2012). Freezing fecal samples prior to DNA extraction affects the Firmicutes to Bacteroidetes ratio determined by downstream quantitative PCR analysis. FEMS Microbiol. Lett..

[44] Ohene-Adjei S., Chaves A.V., McAllister T.A., Benchaar C., Teather R.M., Forster R.J. (2008). Evidence of increased diversity of methanogenic archaea with plant extract supplementation. Microb. Ecol..

[45] Alrawashdeh M., Radwan T. (2017). Wilk’s lambda based on robust method. AIP Conf. Proc..

[46] Liu H., Xu T., Xu S., Ma L., Han X., Wang X., Zhang X., Hu L., Zhao N., Chen Y. (2019). Effect of dietary concentrate to forage ratio on growth performance, rumen fermentation and bacterial diversity of Tibetan sheep under barn feeding on the Qinghai-Tibetan plateau. PeerJ.

[47] Wang L., Li Y., Zhang Y., Wang L. (2020). The Effects of Different Concentrate-to-Forage Ratio Diets on Rumen Bacterial Microbiota and the Structures of Holstein Cows during the Feeding Cycle. Animals.

[48] Vargas J.E., Andrés S., Snelling T.J., López-Ferreras L., Yáñez-Ruíz D.R., García-Estrada C., López S. (2017). Effect of Sunflower and Marine Oils on Ruminal Microbiota, In vitro Fermentation and Digesta Fatty Acid Profile. Front. Microbiol..

[49] Shaani Y., Zehavi T., Eyal S., Miron J., Mizrahi I. (2018). Microbiome niche modification drives diurnal rumen community assembly, overpowering individual variability and diet effects. ISME J..

[50] Franzolin R., Dehority B.A. (1996). Effect of prolonged high-concentrate feeding on ruminal protozoa concentrations. J. Anim. Sci..

[51] Mao S.Y., Huo W.J., Zhu W.Y. (2016). Microbiome-metabolome analysis reveals unhealthy alterations in the composition and metabolism of ruminal microbiota with increasing dietary grain in a goat model. Environ. Microbiol..

[52] Hook S.E., Steele M.A., Northwood K.S., Dijkstra J., France J., Wright A.D., McBride B.W. (2011). Impact of subacute ruminal acidosis (SARA) adaptation and recovery on the density and diversity of bacteria in the rumen of dairy cows. FEMS Microbiol. Ecol..

[53] Zhang J., Shi H., Wang Y., Li S., Cao Z., Ji S., He Y., Zhang H. (2017). Effect of Dietary Forage to Concentrate Ratios on Dynamic Profile Changes and Interactions of Ruminal Microbiota and Metabolites in Holstein Heifers. Front. Microbiol..

[54] Huws S.A., McBain A.J., Gilbert P. (2005). Protozoan grazing and its impact upon population dynamics in biofilm communities. J. Appl. Microbiol..

[55] Tan C., Ramírez-Restrepo C.A., Shah A.M., Hu R., Bell M., Wang Z., McSweeney C. (2020). The community structure and microbial linkage of rumen protozoa and methanogens in response to the addition of tea seed saponins in the diet of beef cattle. J. Anim. Sci. Biotechnol..

[56] Bainbridge M.L., Saldinger L.K., Barlow J.W., Alvez J.P., Roman J., Kraft J. (2018). Alteration of Rumen Bacteria and Protozoa Through Grazing Regime as a Tool to Enhance the Bioactive Fatty Acid Content of Bovine Milk. Front. Microbiol..

[57] Ramos-Vega A., Rosales-Mendoza S., Bañuelos-Hernández B., Angulo C. (2018). Prospects on the Use of *Schizochytrium* sp. to Develop Oral Vaccines. Front. Microbiol..

[58] Tsui C.K., Marshall W., Yokoyama R., Honda D., Lippmeier J.C., Craven K.D., Peterson P.D., Berbee M.L. (2009). Labyrinthulomycetes phylogeny and its implications for the evolutionary loss of chloroplasts and gain of ectoplasmic gliding. Mol. Phylogenet. Evol..

[59] Devillard E., McIntosh F.M., Newbold C.J., Wallace R.J. (2006). Rumen ciliate protozoa contain high concentrations of conjugated linoleic acids and vaccenic acid, yet do not hydrogenate linoleic acid or desaturate stearic acid. Br. J. Nutr..

[60] Sultana H., Miyazawa K., Kanda S., Itabashi H. (2011). Fatty acid composition of ruminal bacteria and protozoa, and effect of defaunation on fatty acid profile in the rumen with special reference to conjugated linoleic acid in cattle. Anim. Sci. J..

[61] Hartinger T., Zebeli Q. (2021). The Present Role and New Potentials of Anaerobic Fungi in Ruminant Nutrition. J. Fungi.

[62] Danielsson R., Dicksved J., Sun L., Gonda H., Muller B., Schnurer A., Bertilsson J. (2017). Methane production in dairy cows correlates with rumen methanogenic and bacterial community structure. Front. Microbiol..

[63] Kittelmann S., Pinares-Patino C.S., Seedorf H., Kirk M.R., Ganesh S., McEwan J.C., Janssen P.H. (2014). Two different bacterial community types are linked with the low methane emission trait in sheep. PLoS ONE.

[64] Shi W., Moon C.D., Leahy S.C., Kang D., Froula J., Kittelmann S., Fan C., Deutsch S., Gagic D., Seedorf H. (2014). Methane yield phenotypes linked to differential gene expression in the sheep rumen microbiome. Genome Res..

[65] McKain N., Shingfield K.J., Wallace R.J. (2010). Metabolism of conjugated linoleic acids and 18:1 fatty acids by 904 ruminal bacteria: Products and mechanisms. Microbiology.

[66] Wallace R.J., Chaudhary L.C., McKain N., McEwan N.R., Richardson A.J., Vercoe P.E., Walker N.D., Paillard D. (2006). Clostridium proteoclasticum: A ruminal bacterium that forms stearic acid from linoleic acid. FEMS Microbiol. Lett..

[67] Dewanckele L., Vlaeminck B., Hernandez-Sanabria E., Ruiz-González A., Debruyne S., Jeyanathan J., Fievez V. (2018). Rumen Biohydrogenation and Microbial Community Changes Upon Early Life Supplementation of 22:6n-3 Enriched Microalgae to Goats. Front. Microbiol..

[68] Huws S.A., Lee M.R., Muetzel S.M., Scott M.B., Wallace R.J., Scollan N.D. (2010). Forage type and fish oil cause shifts in rumen bacterial diversity. FEMS Microbiol. Ecol..

[69] Toral P.G., Belenguer A., Shingfield K.J., Hervás G., Toivonen V., Frutos P. (2012). Fatty acid composition and bacterial community changes in the rumen fluid of lactating sheep fed sunflower oil plus incremental levels of marine algae. J. Dairy Sci..

[70] Zhu H., Fievez V., Mao S., He W., Zhu W. (2016). Dose and time response of ruminally infused algae on rumen fermentation characteristics, biohydrogenation and Butyrivibrio group bacteria in goats. J. Anim. Sci. Biotechnol..

[71] Huws S.A., Kim E.J., Lee M.R., Scott M.B., Tweed J.K., Pinloche E., Wallace R.J., Scollan N.D. (2011). As yet uncultured bacteria phylogenetically classified as Prevotella, Lachnospiraceaeincertae sedis and unclassified Bacteroidales, Clostridiales and Ruminococcaceae may play a predominant role in ruminal biohydrogenation. Environ. Microbiol..

[72] Harfoot G.C., Hazlewood G.P., Hobson P.N., Stewart C.S. (1997). Lipid metabolism in the rumen. The Rumen Microbial Ecosystem.

[73] Huang G., Zhang Y., Xu Q., Zheng N., Zhao S., Liu K., Qu X., Yu J., Wang J. (2020). DHA content in milk and biohydrogenation pathway in rumen: A review. PeerJ.

[74] Liu K., Li Y., Luo G., Xin H., Zhang Y., Li G. (2019). Relations of Ruminal Fermentation Parameters and Microbial Matters to Odd- and Branched-Chain Fatty Acids in Rumen Fluid of Dairy Cows at Different Milk Stages. Animals.

[75] Li R., Teng Z., Lang C., Zhou H., Zhong W., Ban Z., Yan X., Yang H., Farouk M.H., Lou Y. (2019). Effect of different forage-to-concentrate ratios on ruminal bacterial structure and real-time methane production in sheep. PLoS ONE.

[76] Cantalapiedra-Hijar G., Yañez-Ruiz D., Martin-Garcia A., Molina-Alcaide E. (2009). Effects of forage: Concentrate ratio and forage type on apparent digestibility, ruminal fermentation, and microbial growth in goats. J. Anim. Sci. Am. Soc. Anim. Sci..

[77] Hackmann T.J., Firkins J.L. (2015). Maximizing efficiency of rumen microbial protein production. Front. Microbiol..

[78] Zhao X.H., Liu C.J., Liu Y., Li C.Y., Yao J.H. (2013). Effects of replacing dietary starch with neutral detergent–soluble fibre on ruminal fermentation, microbial synthesis and populations of ruminal cellulolytic bacteria using the rumen simulation technique (RUSITEC). J. Anim. Physiol. Anim. Nutr..

[79] Azizi-Shotorkhoft A., Sharifi A., Azarfar A., Kiani A. (2018). Effects of different carbohydrate sources on activity of rumen microbial enzymes and nitrogen retention in sheep fed diet containing recycled poultry bedding. J. Appl. Anim. Res..

[80] Fievez V., Boeckaert C., Vlaeminck B., Mestdagh J., Demeyer D. (2007). In vitro examination of DHA-edible micro-algae. Anim. Feed Sci. Tech..

[81] Morgavi D.P., Forano E., Martin C., Newbold C.J. (2010). Microbial ecosystem and methanogenesis in ruminants. Animal.

[82] Asanuma N., Hino T. (2002). Regulation of fermentation in a ruminal bacterium, *Streptococcus bovis*, with special reference to rumen acidosis. Anim. Sci. J..

[83] Xu N., Wang D., Wang B., Wang J., Liu J. (2019). Different endosperm structures in wheat and corn affected in vitro rumen fermentation and nitrogen utilization of rice straw-based diet. Animal.

