# Supplementary material for: The Effect of Forage-to-Concentrate Ratio on Schizochytrium spp.-Supplemented Goats: Modifying Rumen Microbiota
Source: Animals (Basel). 2021 Sep 20;11(9):2746. doi: 10.3390/ani11092746 (PMC8466047; doi:10.3390/ani11092746)
Supplement: Supplementary file 1 [file animals-11-02746-s001.zip › animals-1327489-supplementary.pdf]

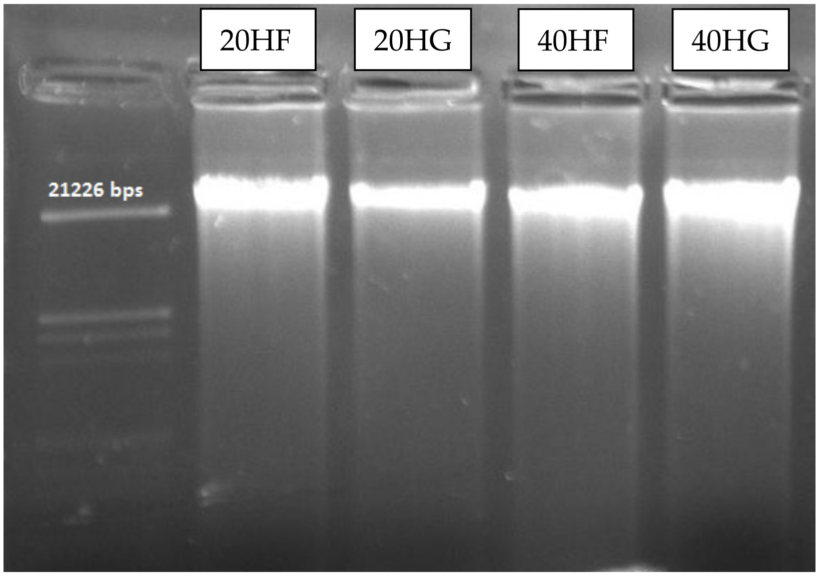

Figure S1. DNA quality of samples used for Ion Torrent.

20HF (22 DNA samples pooled: 11 goats x 2 sampling time): 20g *Schizochytrium spp* and high forage diet (60:40); 20HG (22 DNA samples pooled: 11 goats x 2 sampling time): 20g *Schizochytrium spp* and high grain diet (40:60); 40HF (22 DNA samples pooled: 11 goats x 2 sampling time): 40g *Schizochytrium spp* and high forage diet (60:40); 40HG (22 DNA samples pooled: 11 goats x 2 sampling time): 40g *Schizochytrium spp* and high grain diet (40:60).

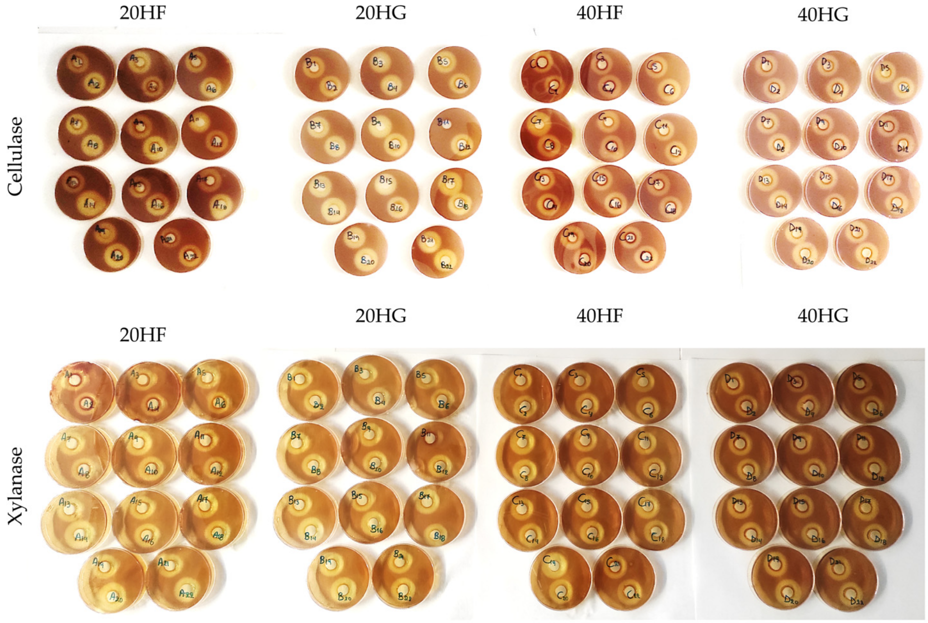

Figure S2. Cellulase and Xylanase enzymatic activity using the petri method.

20HF

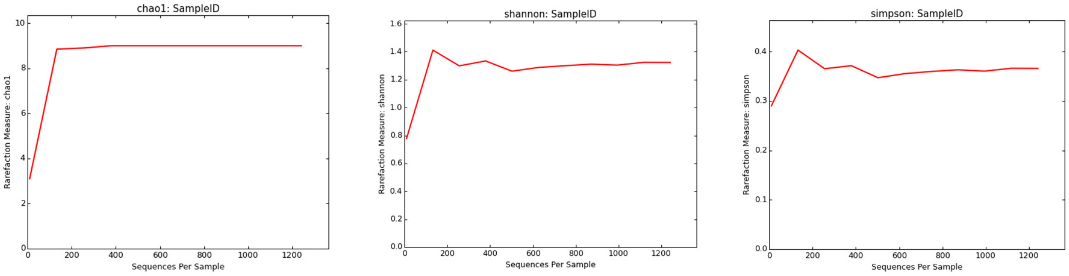

SampleID	Seqs/Sample	chao1 Ave.	chao1 Err.	observed_species Ave.	observed_species Err.	shannon Ave.	shannon Err.	simpson Ave.	simpson Err.
S001_16S002S1_v1	10.0	3.100	nan	2.600	nan	0.778	nan	0.290	nan
S001_16S002S1_v1	133.0	8.850	nan	8.500	nan	1.411	nan	0.403	nan
S001_16S002S1_v1	256.0	8.900	nan	8.900	nan	1.299	nan	0.365	nan
S001_16S002S1_v1	379.0	9.000	nan	9.000	nan	1.334	nan	0.371	nan
S001_16S002S1_v1	502.0	9.000	nan	9.000	nan	1.261	nan	0.347	nan
S001_16S002S1_v1	625.0	9.000	nan	9.000	nan	1.287	nan	0.355	nan
S001_16S002S1_v1	748.0	9.000	nan	9.000	nan	1.300	nan	0.360	nan
S001_16S002S1_v1	871.0	9.000	nan	9.000	nan	1.311	nan	0.363	nan
S001_16S002S1_v1	994.0	9.000	nan	9.000	nan	1.304	nan	0.360	nan
S001_16S002S1_v1	1117.0	9.000	nan	9.000	nan	1.324	nan	0.366	nan
S001_16S002S1_v1	1240.0	9.000	nan	9.000	nan	1.323	nan	0.366	nan

20HG

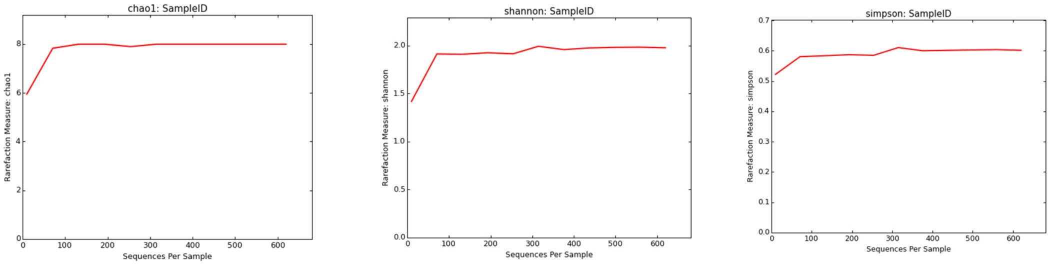

SampleID	Seqs/Sample	chao1 Ave.	chao1 Err.	observed_species Ave.	observed_species Err.	shannon Ave.	shannon Err.	simpson Ave.	simpson Err.
S001_16S002S2_v1	10.0	5.950	nan	3.800	nan	1.419	nan	0.522	nan
S001_16S002S2_v1	71.0	7.833	nan	7.800	nan	1.914	nan	0.581	nan
S001_16S002S2_v1	132.0	8.000	nan	8.000	nan	1.910	nan	0.584	nan
S001_16S002S2_v1	193.0	8.000	nan	8.000	nan	1.926	nan	0.587	nan
S001_16S002S2_v1	254.0	7.900	nan	7.900	nan	1.915	nan	0.585	nan
S001_16S002S2_v1	315.0	8.000	nan	8.000	nan	1.994	nan	0.610	nan
S001_16S002S2_v1	376.0	8.000	nan	8.000	nan	1.959	nan	0.600	nan
S001_16S002S2_v1	437.0	8.000	nan	8.000	nan	1.976	nan	0.601	nan
S001_16S002S2_v1	498.0	8.000	nan	8.000	nan	1.983	nan	0.603	nan
S001_16S002S2_v1	559.0	8.000	nan	8.000	nan	1.984	nan	0.604	nan
S001_16S002S2_v1	620.0	8.000	nan	8.000	nan	1.978	nan	0.602	nan

40HF

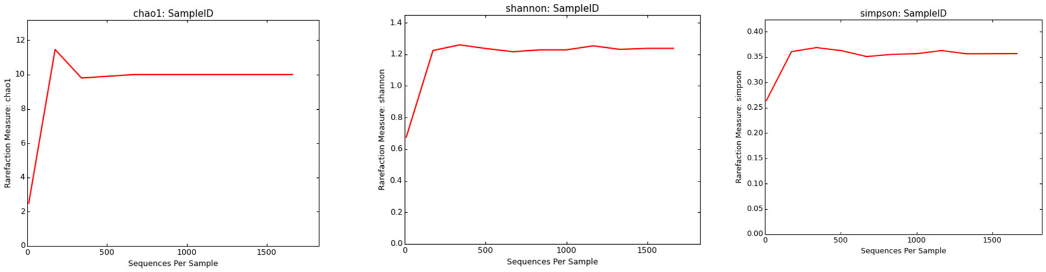

SampleID	Seqs/Sample	chao1 Ave.	chao1 Err.	observed_species Ave.	observed_species Err.	shannon Ave.	shannon Err.	simpson Ave.	simpson Err.
S001_16S002S3_v1	10.0	2.500	nan	2.300	nan	0.676	nan	0.264	nan
S001_16S002S3_v1	175.0	11.462	nan	9.700	nan	1.224	nan	0.360	nan
S001_16S002S3_v1	340.0	9.800	nan	9.800	nan	1.260	nan	0.368	nan
S001_16S002S3_v1	505.0	9.900	nan	9.900	nan	1.236	nan	0.362	nan
S001_16S002S3_v1	670.0	10.000	nan	10.000	nan	1.216	nan	0.351	nan
S001_16S002S3_v1	835.0	10.000	nan	10.000	nan	1.228	nan	0.355	nan
S001_16S002S3_v1	1000.0	10.000	nan	10.000	nan	1.228	nan	0.356	nan
S001_16S002S3_v1	1165.0	10.000	nan	10.000	nan	1.253	nan	0.363	nan
S001_16S002S3_v1	1330.0	10.000	nan	10.000	nan	1.231	nan	0.356	nan
S001_16S002S3_v1	1495.0	10.000	nan	10.000	nan	1.237	nan	0.356	nan
S001_16S002S3_v1	1660.0	10.000	nan	10.000	nan	1.238	nan	0.356	nan

40HG

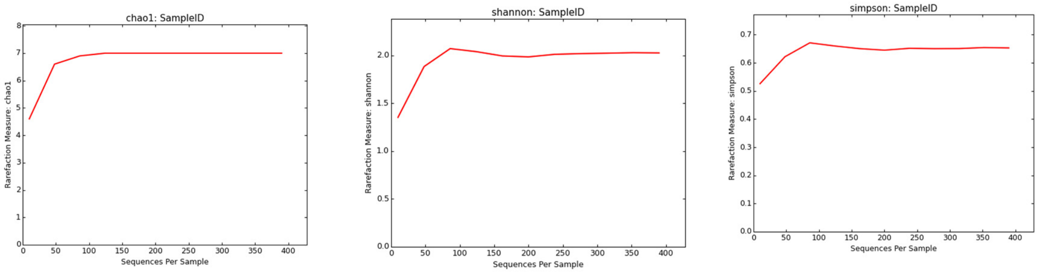

SampleID	Seqs/Sample	chao1 Ave.	chao1 Err.	observed_species Ave.	observed_species Err.	shannon Ave.	shannon Err.	simpson Ave.	simpson Err.
S001_16S002S4_v1	10.0	4.600	nan	3.400	nan	1.353	nan	0.526	nan
S001_16S002S4_v1	48.0	6.600	nan	6.400	nan	1.884	nan	0.622	nan
S001_16S002S4_v1	86.0	6.900	nan	6.900	nan	2.072	nan	0.671	nan
S001_16S002S4_v1	124.0	7.000	nan	7.000	nan	2.040	nan	0.660	nan
S001_16S002S4_v1	162.0	7.000	nan	7.000	nan	1.995	nan	0.651	nan
S001_16S002S4_v1	200.0	7.000	nan	7.000	nan	1.985	nan	0.645	nan
S001_16S002S4_v1	238.0	7.000	nan	7.000	nan	2.012	nan	0.652	nan
S001_16S002S4_v1	276.0	7.000	nan	7.000	nan	2.020	nan	0.650	nan
S001_16S002S4_v1	314.0	7.000	nan	7.000	nan	2.024	nan	0.651	nan
S001_16S002S4_v1	352.0	7.000	nan	7.000	nan	2.029	nan	0.654	nan
S001_16S002S4_v1	390.0	7.000	nan	7.000	nan	2.027	nan	0.653	nan

Figure S3. Rarefaction curves and alpha diversity of the four dietary groups (20HF, 20HG, 40HF, and 40HG) at genera taxonomic level.

20HF (22 DNA samples pooled: 11 goats x 2 sampling time): 20g *Schizochytrium spp* and high forage diet (60:40); 20HG (22 DNA samples pooled: 11 goats x 2 sampling time): 20g *Schizochytrium spp* and high grain diet (40:60); 40HF (22 DNA samples pooled: 11 goats x 2 sampling time): 40g *Schizochytrium spp* and high forage diet (60:40); 40HG (22 DNA samples pooled: 11 goats x 2 sampling time): 40g *Schizochytrium spp* and high grain diet (40:60).

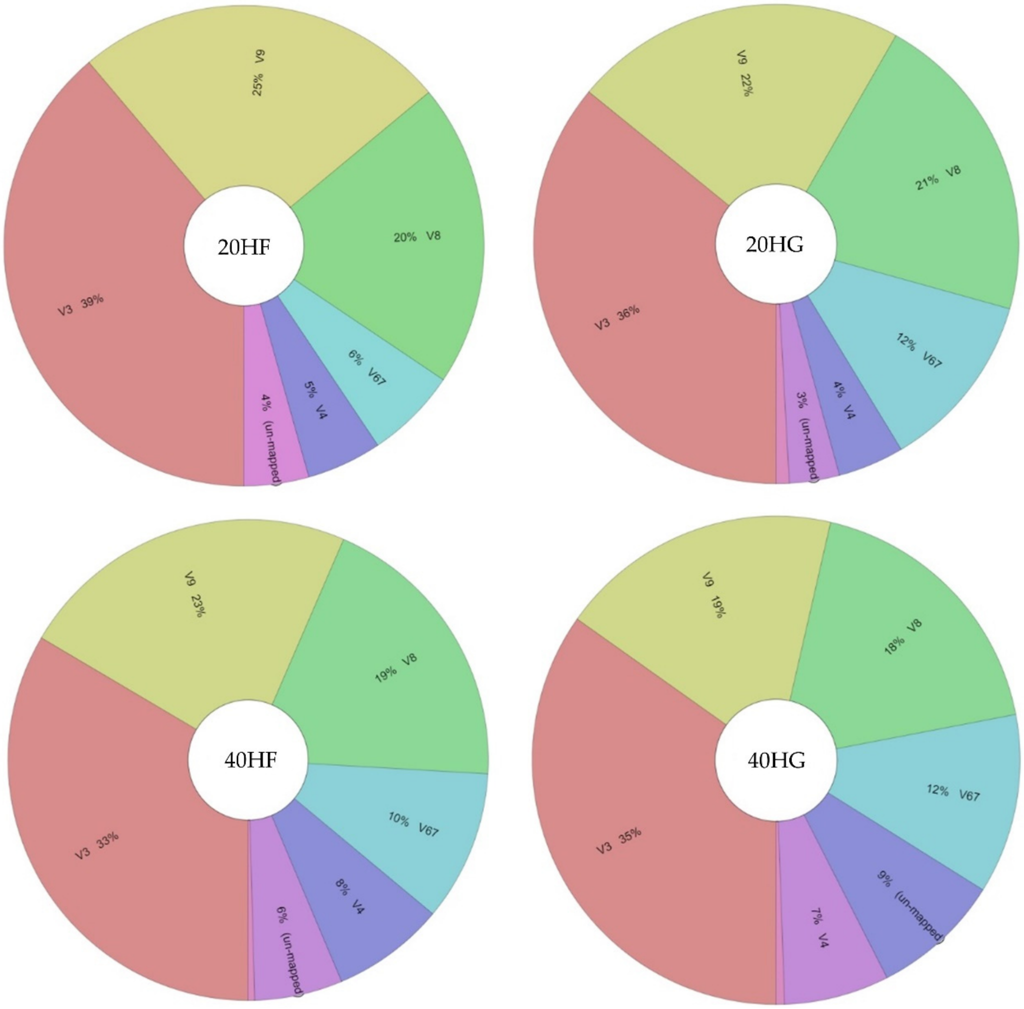

Figure S4. Primers coverage in the four samples of goats rumen.
20HF (22 DNA samples pooled: 11 goats x 2 sampling time): 20g *Schizochytrium spp* and high forage diet (60:40); 20HG (22 DNA samples pooled: 11 goats x 2 sampling time): 20g *Schizochytrium spp* and high grain diet (40:60); 40HF (22 DNA samples pooled: 11 goats x 2 sampling time): 40g *Schizochytrium spp* and high forage diet (60:40); 40HG (22 DNA samples pooled: 11 goats x 2 sampling time): 40g *Schizochytrium spp* and high grain diet (40:60).

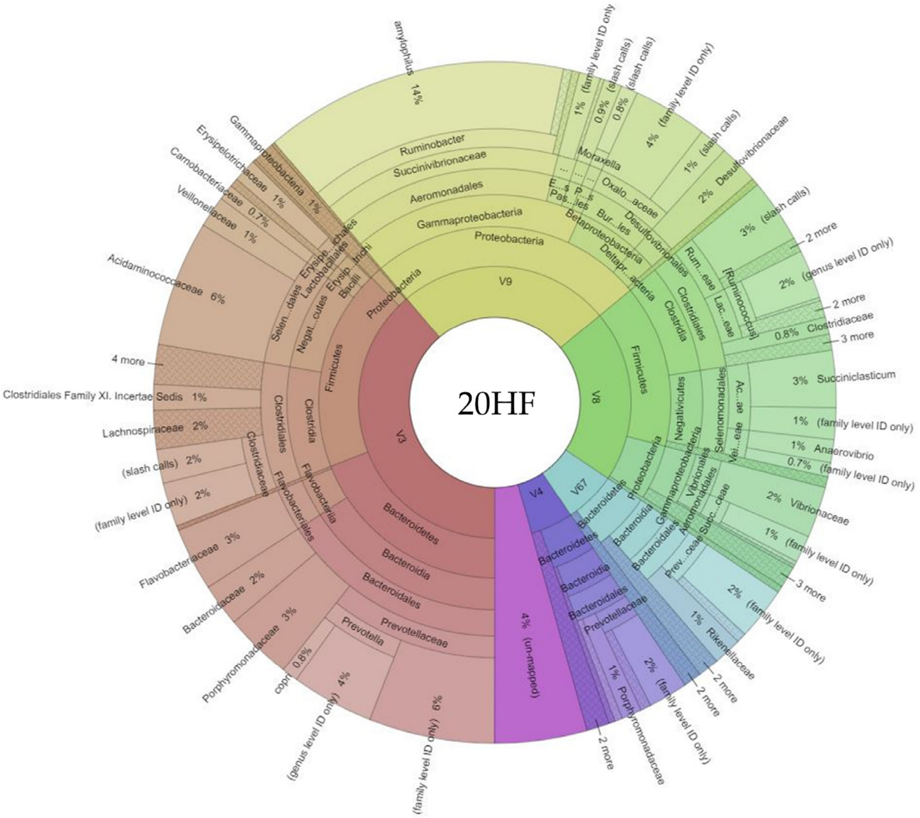
20HF
V9
V8
V3
V4
V67
Proteobacteria
Gammaproteobacteria
Aeromonadales
Succinivibrionaceae
Ruminobacter
amylophilus 14%
Betaproteobacteria
Firmicutes
Clostridia
Clostridiales
Negativicutes
Selenomonadales
Bacteroidetes
Bacteroidia
Bacteroidales
Prevotellaceae
Prevotella
Flavobacteria
Flavobacteriales
4% (un-mapped)
Acidaminococcaceae 6%
Clostridiales Family XI. Incertae Sedis 1%
Lachnospiraceae 2%
Flavobacteriaceae 3%
Bacteroidaceae 2%
Porphyromonadaceae 3%
3% Succiniclastium
2% Vibrionaceae
1% Anaerovibrio
Rikenellaceae
Desulfovibrionaceae

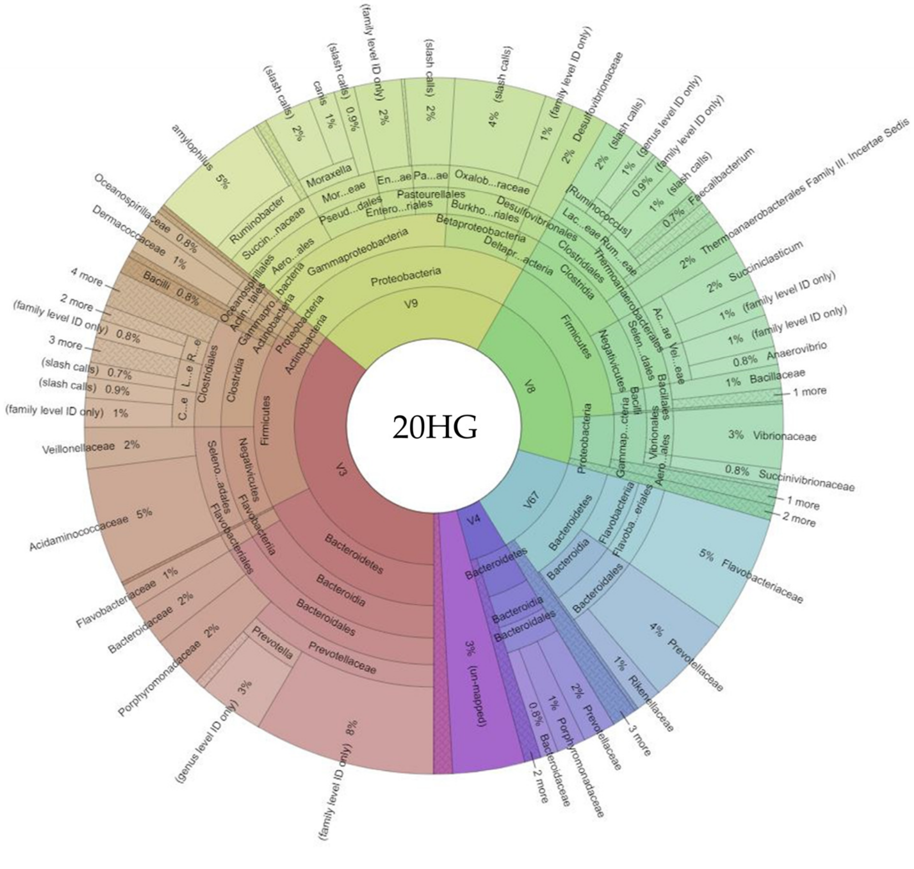
20HG
V9
V8
V3
V4
V67
Proteobacteria
Gammaproteobacteria
Betaproteobacteria
amylophilus 5%
Firmicutes
Clostridia
Clostridiales
Negativicutes
Bacteroidetes
Bacteroidia
Bacteroidales
Prevotellaceae
Prevotella
Flavobacteria
Flavobacteriales
3% (un-mapped)
Veillonellaceae 2%
Acidaminococcaceae 5%
Flavobacteriaceae 1%
Bacteroidaceae 2%
Porphyromonadaceae 2%
5% Flavobacteriaceae
4% Prevotellaceae
3% Vibrionaceae
2% Succiniclastium
0.8% Anaerovibrio
1% Bacillaceae
0.8% Succinivibrionaceae
Thermoanaerobacterales Family III. Incertae Sedis
Faecalibacterium
Rikenellaceae

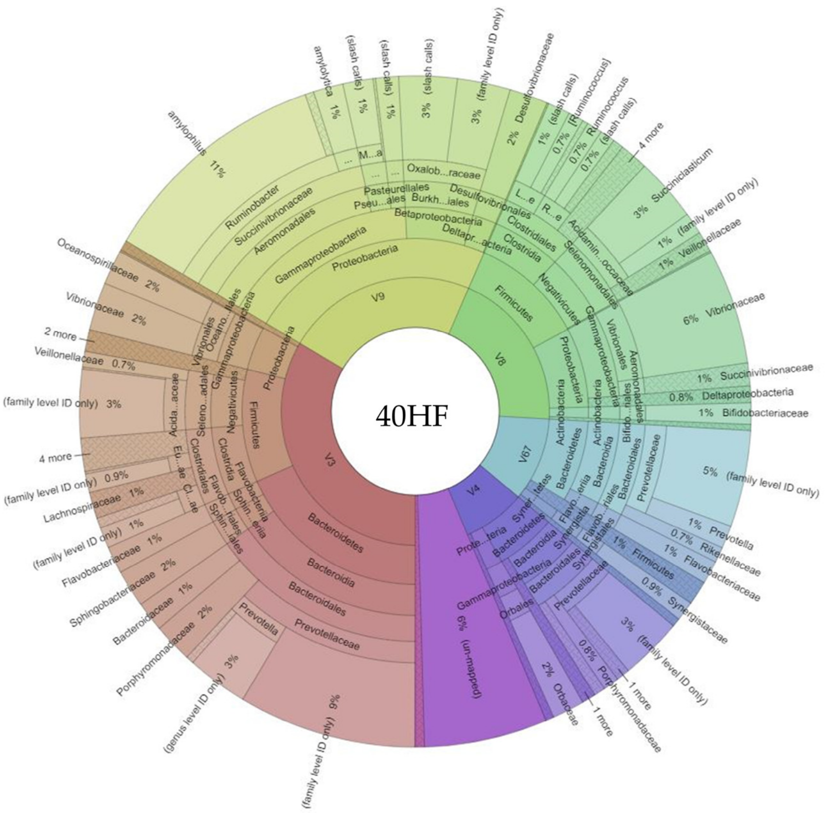

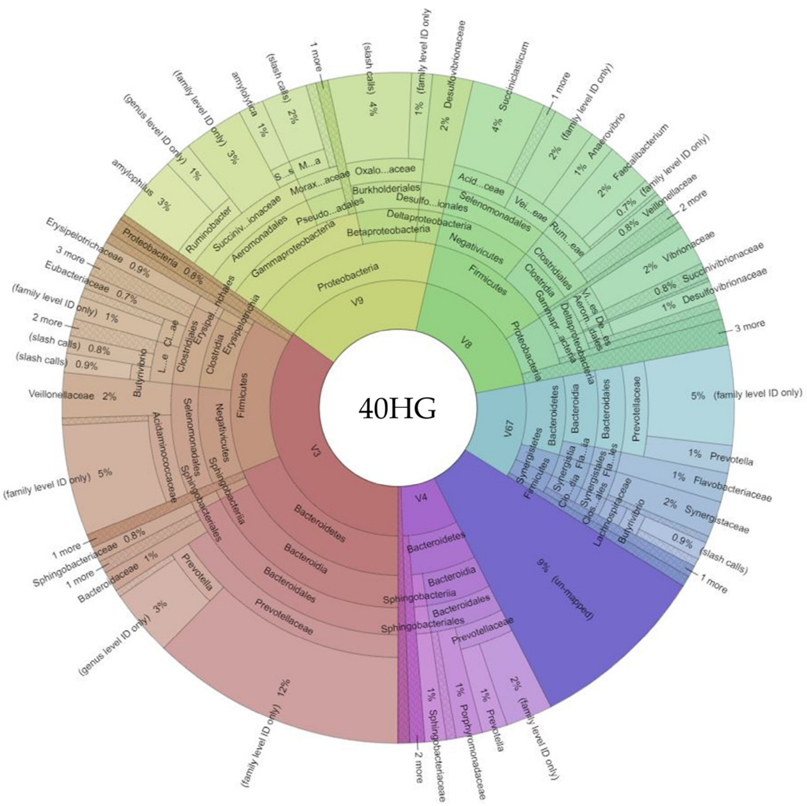

Figure S5. Metagenomic visualization using Krona software are available in family level.

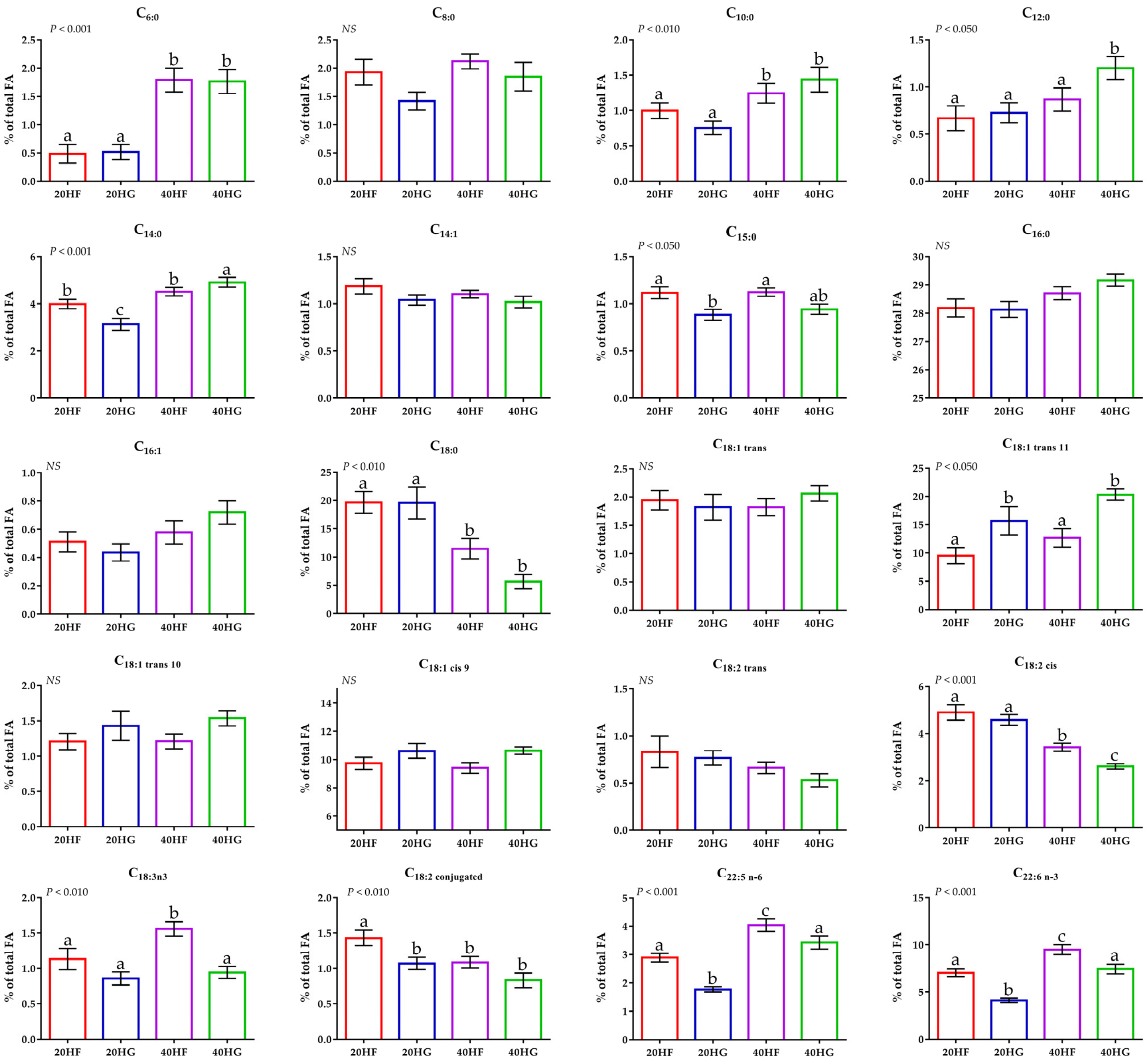

Figure S6. The mean individual fatty acids (FA) (% of total FA) in rumen fluid of goats fed diets (20HF, 20HG, 40HF, and 40HG) with different levels of *Schizochytrium spp.* (20 g and 40 g/goat/day) and two different forage to concentrate ratios (60:40 and 40:60) throughout the experimental period (21 and 42 experimental days) illustrated in column bars (±Standard Error of Means). Bars with different superscript (a, b, c) between dietary treatments differ significantly ($p \leq 0.05$) while t is referred to P-value <0.10, according to the Analysis of Variance (ANOVA) using a general linear model for repeated measures and Post hoc analysis was performed when appropriate using Tukey's multiple range test.

20HF (n = 11 goats): 20g *Schizochytrium spp* and high forage diet (60:40); 20HG (n = 11 goats): 20g *Schizochytrium spp* and high grain diet (40:60); 40HF (n = 11 goats): 40g *Schizochytrium spp* and high forage diet (60:40); 40HG (n = 11 goats): 40g *Schizochytrium spp* and high grain diet (40:60).

Table S1. Ingredients of concentrate (g/Kg) of the four diets.

	Concentrates			
	**20HF**	**20HG**	**40HF**	**40HG**
Maize grain	387	331.6	357	326.3
Barley grain	200	200	200	200
Wheat middlings	210	210	210	210
Sunflower meal,	80	80	80	80
Soybean meal	60	120	70	110
Calcium phosphate	15	15	15	15
Calcium carbonate	5	5	5	5
Salt	3	3	3	3
Mineral and vitamin	20	20	20	20
Schizochytrium spp. (g)	20	15.4	40	30.7

20HF: 20g *Schizochytrium spp* and high forage diet (60:40); 20HG: 20g *Schizochytrium spp* and high grain diet (40:60); 40HF: 40g *Schizochytrium spp* and high forage diet (60:40); 40HG: 40g *Schizochytrium spp* and high grain diet (40:60)

Table S2. Feed chemical composition (%).

	Concentrates				Forages	
	**20HF**	**20HG**	**40HF**	**40HG**	**Alfalfa hay**	**Wheat straw**
Dry Matter	90.8	91.0	92.4	91.8	91.1	93.7
Ash	4.1	4.5	4.5	4.3	10.3	7.7
Crude Protein	13.6	16	13.6	16	14	2.5
Ether Extract	6.1	5.8	6.9	6.5	1.3	1.9
Ash-free NDF treated with amylase	21.9	22.5	21.8	22.4	41.5	71.5
Acid Detergent Fiber	7.2	8.9	8.0	8.6	30.6	50.7
Non Fibrous Carbohydrate	54.3	51.2	53.2	50.8	32.9	16.4
Starch	43.1	39.8	41.9	39.8	3.3	1.0

20HF: 20g *Schizochytrium spp* and high forage diet (60:40); 20HG: 20g *Schizochytrium spp* and high grain diet (40:60); 40HF: 40g *Schizochytrium spp* and high forage diet (60:40); 40HG: 40g *Schizochytrium spp* and high grain diet (40:60)

Table S3. Relative abundance of rumen fluid bacteriome of goats fed diets (20HF, 20HG, 40HF and 40HG) with different levels of *Schizochytrium spp.* (20 g and 40 g/goat/day) and two different forage to concentrate ratios (60:40 and 40:60) throughout the experimental period (21 and 42 experimental days) using Ion-Torrent sequencing.

**Phylum**	**Class**	**Order**	**Family**	**Genus**	**Species**	**20HF**	**20HG**	**40HF**	**40HG**
						100	100	100	100
Actinobacteria						0.51	1.79	1.6	0.87
	Actinobacteria					0.51	1.79	1.6	0.87
		Actinomycetales				0.17	1.39	0.39	0.16
			Corynebacteriaceae			N.D.	N.D.	0.15	N.D.
			Dermacoccaceae			0.17	1.39	0.24	0.16
		Bifidobacteriales				0.34	0.4	1.21	0.7
			Bifidobacteriaceae			0.34	0.4	1.21	0.7
				Bifidobacterium		N.D.	N.D.	0.1	N.D.
Bacteroidetes						29.66	33.75	34.08	36.5
	Bacteroidia					24.94	26.03	28.87	31.95
		Bacteroidales				24.94	26.03	28.87	31.95
			Bacteroidaceae			2.5	2.82	2.11	1.34
			Marinilabiliaceae			N.D.	N.D.	N.D.	0.23
			Porphyromonadaceae			4.35	3.9	2.82	2.03
			Prevotellaceae			16.68	18.08	23.14	28.35
				Prevotella		5.89	3.62	5.38	6.71
					copri	0.8	0.46	0.4	0.36
			Rikenellaceae			1.41	1.23	0.79	N.D.
	Cytophagia					0.15	0.22	N.D.	N.D.
		Cytophagales				0.15	0.22	N.D.	N.D.
			Cytophagaceae			0.15	0.22	N.D.	N.D.
	Flavobacteriia					3.86	7.16	2.58	2.59
		Flavobacteriales				3.86	7.16	2.58	2.59
			Flavobacteriaceae			3.86	7.16	2.58	2.59
	Sphingobacteriia					0.71	0.34	2.63	1.96
		Sphingobacteriales				0.71	0.34	2.63	1.96
			Sphingobacteriaceae			0.71	0.34	2.63	1.96
Firmicutes						36.30	33.66	24.1	32.30

Bacilli					1.36	2.58	0.83	0.83
	Bacillales				0.58	1.97	0.25	0.83
		Bacillaceae			0.32	1.13	0.13	0.16
		Staphylococcaceae			0.26	0.85	0.12	0.67
	Lactobacillales				0.78	0.61	0.58	N.D.
		Carnobacteriaceae			0.78	0.61	0.58	N.D.
Clostridia					19.19	16.83	12.49	13.21
	Clostridiales				19.19	15.1	12.49	13.21
		Christensenellaceae			0.64	0.19	0.36	0.34
		Clostridiaceae			5.24	2.54	2.11	1.67
		Clostridiales Family XI. Incertae Sedis			1.27	0.64	0.64	0.44
		Eubacteriaceae			0.49	0.52	1.38	0.93
		Gracilibacteraceae			0.17	0.34	0.11	N.D.
		Heliobacteriaceae			N.D.	N.D.	N.D.	0.26
		Lachnospiraceae			6.04	6.01	3.92	4.86
			Butyrivibrio		1.1	0.25	0.66	2.39
				fibrisolvens	0.35	N.D.	0.27	0.44
				proteoclasticus	0.58	N.D.	0.17	N.D.
			Coprococcus		0.64	0.4	0.24	0.29
				eutactus	0.64	0.4	0.24	0.29
			Lachnospira		0.2	N.D.	N.D.	N.D.
			[Ruminococcus]		2.59	1.16	0.78	0.38
				gnavus	0.17	0.15	N.D.	N.D.
		Oscillospiraceae			0.32	0.21	0.42	N.D.
		Peptococcaceae			0.17	N.D.	0.1	N.D.
		Ruminococcaceae			4.54	4.29	2.83	3.81
			Faecalibacterium		0.2	0.7	0.45	2.37
				prausnitzii	0.2	0.53	0.24	N.D.
			Ruminococcus		1.18	0.74	1.28	0.16
				flavefaciens	0.84	0.36	0.11	0.16
		Veillonellaceae			0.31	0.37	0.62	0.88
	Thermoanaerobacterales				N.D.	1.74	N.D.	N.D.
		Thermoanaerobacterales Family III. Incertae Sedis			N.D.	1.74	N.D.	N.D.
Erysipelotrichi					1.55	0.47	0.54	0.61

		Erysipelotrichales			1.55	0.47	0.54	0.61
			Erysipelotrichaceae		1.55	0.47	0.54	0.61
	Erysipelotrichia				0.15	N.D.	0.41	0.93
		Erysipelotrichales			0.15	N.D.	0.41	0.93
			Erysipelotrichaceae		0.15	N.D.	0.41	0.93
	Negativicutes				14.04	13.78	9.83	16.71
		Selenomonadales			14.04	13.78	9.83	16.71
			Acidaminococcaceae		10.64	8.85	7.97	10.79
				Succiniclasticum	2.82	1.97	3.36	4.26
				ruminis	N.D.	N.D.	0.11	0.36
			Veillonellaceae		3.4	4.92	1.86	5.93
				Anaerovibrio	1.13	0.87	0.38	1.31
				Selenomonas	N.D.	N.D.	0.13	N.D.
Proteobacteria					33.34	29.93	38.82	27.25
	Alphaproteobacteria				0.61	N.D.	0.09	0.56
		Sphingomonadales			0.61	N.D.	0.09	0.38
			Erythrobacteraceae		0.61	N.D.	0.09	0.38
		Sneathiellales			N.D.	N.D.	N.D.	0.18
			Sneathiellaceae		N.D.	N.D.	N.D.	0.18
	Betaproteobacteria				5.1	5.74	5.8	5.63
		Burkholderiales			5.1	5.74	5.69	5.47
			Oxalobacteraceae		5.1	5.74	5.69	5.47
		Neisseriales			N.D.	N.D.	N.D.	0.16
			Neisseriaceae		N.D.	N.D.	N.D.	0.16
		Rhodocyclales			N.D.	N.D.	0.11	N.D.
			Rhodocyclaceae		N.D.	N.D.	0.11	N.D.
	Deltaproteobacteria				2.61	2.73	2.92	4.35
		Desulfovibrionales			2.13	2.58	2.6	3.86
			Desulfovibrionaceae		2.13	2.58	2.6	3.86
				Desulfovibrio	N.D.	N.D.	0.09	N.D.
				D168	N.D.	N.D.	0.09	N.D.
		Syntrophobacterales			0.48	0.15	0.31	0.49
			Syntrophaceae		0.48	0.15	0.31	0.49
	Epsilonproteobacteria				0.18	N.D.	N.D.	N.D.

		Campylobacterales				0.18	N.D.	N.D.	N.D.
			Campylobacteraceae			0.18	N.D.	N.D.	N.D.
	Gammaproteobacteria					24.83	21.46	30.01	16.71
		Aeromonadales				17.41	7.06	15.26	10.44
			Succinivibrionaceae			17.41	7.06	15.25	10.44
				Ruminobacter		15.5	5.78	12.66	4.86
					amylophilus	15.07	5.61	12.21	3.44
				Succinimonas		0.37	0.49	1.52	1.33
					amylolytica	0.37	0.49	1.52	1.33
				Succinivibrio		0.2	N.D.	0.11	0.39
		Enterobacteriales				1.47	2.4	0.14	N.D.
			Enterobacteriaceae			1.47	2.4	0.14	N.D.
		Oceanospirillales				0.66	0.8	1.78	N.D.
			Oceanospirillaceae			0.66	0.8	1.78	N.D.
		Orbales				0.43	0.21	1.64	N.D.
			Orbaceae			0.43	0.21	1.64	N.D.
		Pasteurellales				0.89	2.65	1.17	0.64
			Pasteurellaceae			0.89	2.65	1.17	0.64
		Pseudomonadales				0.84	4.64	1.67	2.93
			Moraxellaceae			0.84	4.64	1.67	2.93
				Moraxella		0.84	3.49	1.54	2.41
					canis	N.D.	1.25	N.D.	N.D.
		Vibrionales				2.97	3.34	8.35	2.54
			Vibrionaceae			2.97	3.34	8.35	2.54
		Xanthomonadales				0.15	0.36	N.D.	0.16
			Xanthomonadaceae			0.15	0.36	N.D.	0.16
Spirochaetes						N.D.	N.D.	N.D.	0.16
	Spirochaetia					N.D.	N.D.	N.D.	0.16
		Spirochaetales				N.D.	N.D.	N.D.	0.16
			Spirochaetaceae			N.D.	N.D.	N.D.	0.16
Synergistetes						0.2	0.86	1.15	2.5
	Synergistia					0.2	0.86	1.15	2.5
		Synergistales				0.2	0.86	1.15	2.5
			Dethiosulfovibrionaceae			N.D.	N.D.	N.D.	0.36

			Synergistaceae	0.2	0.86	1.15	2.14
Tenericutes				N.D.	N.D.	0.14	N.D.
	Mollicutes			N.D.	N.D.	0.14	N.D.
		Entomoplasmatales		N.D.	N.D.	0.14	N.D.
			Entomoplasmataceae	N.D.	N.D.	0.14	N.D.
Verrucomicrobia				N.D.	N.D.	0.11	0.41
	Verrucomicrobiae			N.D.	N.D.	0.11	0.41
		Verrucomicrobiales		N.D.	N.D.	0.11	0.23
			Verrucomicrobiaceae	N.D.	N.D.	0.11	0.23
		Puniceicoccales		N.D.	N.D.	N.D.	0.18
			Puniceicoccaceae	N.D.	N.D.	N.D.	0.18

20HF (22 DNA samples pooled: 11 goats x 2 sampling time): 20g *Schizochytrium spp* and high forage diet (60:40); 20HG (22 DNA samples pooled: 11 goats x 2 sampling time): 20g *Schizochytrium spp* and high grain diet (40:60); 40HF (22 DNA samples pooled: 11 goats x 2 sampling time): 40g *Schizochytrium spp* and high forage diet (60:40); 40HG (22 DNA samples pooled: 11 goats x 2 sampling time): 40g *Schizochytrium spp* and high grain diet (40:60).

Table S4. Relative abundance of several microorganisms (to the total bacterial 16s DNA) in the rumen fluid of goats fed diets (20HF, 20HG, 40HF, and 40HG) with different levels of microalgae *Schizochytrium* spp (20 g and 40 g/day/goat) and two different forage to concentrate ratios (60:40 and 40:60) throughout the experimental period (21th and 42th experimental day) using a GLM model for repeated measure.

	**Dietary treatment (D)**					**Sampling time (S)**			**Effect**		
	20HF	20HG	40HF	40HG	SEM	21	42	SEM	D	T	D x T
Bacteroidetes	0.355^{a}	0.270^{a}	0.355^{a}	0.445^{b}	0.032	0.319^{a}	0.393^{b}	0.021	**	*	NS
Firmicutes	0.361	0.291	0.284	0.302	0.035	0.263^{a}	0.356^{b}	0.025	NS	*	*
Archaea	0.011	0.013	0.010	0.014	0.002	0.011	0.013	0.001	NS	NS	***
Protozoa	0.006^{b}	0.007^{a}	0.005^{b}	0.006^{b}	<0.001	0.007	0.005	<0.001	**	*	NS
Entodinium spp	0.003^{b}	0.005^{a}	0.005^{ab}	0.006^{a}	0.001	0.006	0.004	0.001	*	NS	t
Total fungi	0.0002	0.0002	0.0002	0.00012	<0.001	0.00017	0.00019	<0.001	NS	NS	NS
Neocallimastigales	0.00014^{b}	0.00012^{b}	0.00019^{a}	0.00012^{b}	<0.001	0.00014	0.00014	<0.001	*	NS	NS
Methanogen	0.005^{a}	0.006^{a}	0.010^{b}	0.008^{ab}	0.001	0.009^{a}	0.005^{b}	0.001	*	**	NS
Methanomassiliicoccales	0.0017^{a}	0.0015^{a}	0.0025^{b}	0.0025^{b}	<0.001	0.0025^{a}	0.0015^{b}	<0.001	*	***	NS
Eubacterium ruminantium	0.0029^{a}	0.0016^{ab}	0.0019^{a}	0.0005^{b}	0.001	0.0015	0.0020	<0.001	*	NS	*
Butyrivibrio fibrisolvens	0.055^{a}	0.059^{a}	0.040^{b}	0.039^{b}	0.005	0.037^{a}	0.059^{b}	0.003	**	***	*
Butyrivibrio proteoclasticus	0.017	0.015	0.010	0.012	0.003	0.011	0.015	0.001	NS	*	NS
Ruminococcus flavefaciens	0.0016^{a}	0.0008^{ab}	0.0006^{b}	0.0005^{b}	<0.001	0.0008	0.0010	<0.001	*	NS	NS
Fibrobacter succinogenes	0.014	0.009	0.018	0.013	0.003	0.016^{a}	0.011^{b}	0.002	NS	*	NS
Ruminococcus albus	0.0005	0.0003	0.0002	0.0002	<0.001	0.0003	0.0003	<0.001	NS	NS	t
Ruminobacter amylophilus	0.030^{t}	0.018^{a}	0.063^{b}	0.027^{t}	0.015	0.045^{a}	0.024^{b}	0.007	*	*	NS
Streptococcus bovis	0.000004^{a}	0.0000035^{a}	0.000001^{b}	0.000001^{b}	<0.001	0.0000025	0.0000023	<0.001	*	NS	NS
Selenomonas ruminantium	0.011	0.011	0.009	0.012	0.001	0.010	0.011	0.001	NS	NS	**
Prevotella spp	0.118	0.109	0.112	0.136	0.011	0.105^{a}	0.133^{b}	0.007	NS	**	NS
Prevotella ruminicola	0.054^{ab}	0.041^{a}	0.042^{a}	0.064^{b}	0.006	0.044^{a}	0.056^{b}	0.004	*	t	NS
Prevotella brevis	0.025^{a}	0.025^{a}	0.058^{b}	0.053^{b}	0.008	0.033	0.047	0.005	**	t	NS
Methanosphaera stadtmanae	0.0011	0.0012	0.00095	0.00081	<0.001	0.00078^{a}	0.0012^{b}	<0.001	NS	**	*
Methanobrevibacter spp.	0.0020^{a}	0.0025^{a}	0.0006^{b}	0.0009^{b}	<0.001	0.0006^{a}	0.0024^{b}	<0.001	**	***	**
Methanobacteriales	0.0016^{a}	0.0024^{b}	0.0008^{c}	0.0015^{a}	<0.001	0.0009^{a}	0.0022^{b}	<0.001	***	***	***

Means with different superscript letters (a, b) between dietary groups and sampling time differ significantly; * $p < 0.05$, ** $p < 0.01$, *** $p < 0.001$, t = trend $p < 0.10$.

[a] SEM: Standard error of the means. 20HF (n = 11 goats): 20g *Schizochytrium spp* and high forage diet (60:40); 20HG (n = 11 goats): 20g *Schizochytrium spp* and high grain diet (40:60); 40HF (n = 11 goats): 40g *Schizochytrium spp* and high forage diet (60:40); 40HG (n = 11 goats): 40g *Schizochytrium spp* and high grain diet (40:60).

Table S5. Relative abundance of several microorganisms several microorganisms (to the total bacterial 16s DNA) in the rumen fluid of goats fed diets (20HF, 20HG, 40HF, and 40HG) with different levels of microalgae Schizochytrium spp (20 g and 40 g/day/goat) and two different forage to concentrate ratios (60:40 and 40:60) throughout the experimental period (21th and 42th experimental day) using a GLM model for three-way repeated measures.

	**Dietary treatment (D)**						**Sampling time (S)**			**Effect**						
	Forage/Concentrate			Algae level			Sampling day			Effect			Interaction effect			
	60/40	40/60	SEM[a]	20g	40g	SEM[a]	21	42	SEM[a]	F/C	ALG	TIME	F/C×A	F/C×T	A×T	F/C×A×T
Bacteroidetes	0.354	0.358	0.024	0.312	0.400	0.032	0.321	0.391	0.035	NS	**	t	**	NS	NS	NS
Firmicutes	0.324	0.297	0.029	0.326	0.295	0.036	0.266	0.355	0.037	NS	NS	*	NS	NS	**	NS
Archaea	0.011	0.013	0.002	0.012	0.012	0.002	0.011	0.013	0.002	NS	NS	NS	NS	NS	***	NS
Protozoa	0.006	0.007	0.0003	0.007	0.006	0.0005	0.007	0.006	0.0005	*	NS	t	NS	NS	NS	NS
Entodinium spp	0.004	0.006	0.001	0.004	0.005	0.001	0.006	0.004	0.001	NS	NS	t	NS	NS	*	NS
Total fungi	2×10^{-4}	2×10^{-4}	2×10^{-5}	2×10^{-4}	2×10^{-4}	2×10^{-5}	2×10^{-4}	2×10^{-4}	3×10^{-5}	NS	NS	NS	NS	NS	NS	NS
Neocallimastigales	2×10^{-4}	1×10^{-4}	2×10^{-5}	1×10^{-4}	2×10^{-4}	2×10^{-5}	1×10^{-4}	1×10^{-4}	2×10^{-5}	t	t	NS	NS	NS	*	NS
Methanogen	0.008	0.007	0.001	0.005	0.009	0.001	0.009	0.005	0.001	NS	**	**	NS	NS	NS	NS
Methanomassiliicoccales	0.002	0.002	0.0002	0.002	0.003	0.0003	0.003	0.002	0.0003	NS	***	***	NS	NS	NS	NS
Eubacterium ruminantium	0.002	0.001	0.0004	0.002	0.001	0.0005	0.002	0.002	0.0005	*	*	NS	NS	NS	**	NS
Butyrivibrio fibrisolvens	0.047	0.053	0.004	0.058	0.041	0.005	0.039	0.060	0.005	NS	***	***	NS	NS	***	NS
Butyrivibrio proteoclasticus	0.013	0.013	0.002	0.016	0.011	0.003	0.011	0.015	0.002	NS	*	*	NS	NS	**	NS
Ruminococcus flavefaciens	9×10^{-4}	7×10^{-4}	2×10^{-4}	1.3×10^{-3}	3×10^{-4}	2×10^{-4}	5×10^{-4}	1×10^{-3}	3×10^{-4}	NS	***	*	NS	NS	*	NS
Fibrobacter succinogenes	0.016	0.011	0.003	0.011	0.016	0.003	0.016	0.012	0.003	t	*	t	NS	NS	NS	NS
Ruminococcus albus	5×10^{-4}	3×10^{-4}	1×10^{-4}	5×10^{-4}	3×10^{-4}	1×10^{-4}	3×10^{-4}	5×10^{-4}	1×10^{-4}	t	*	*	NS	*	**	NS
Ruminobacter amylophilus	0.046	0.023	0.010	0.025	0.044	0.014	0.044	0.025	0.012	NS	NS	t	NS	NS	NS	NS
Streptococcus bovis	2×10^{-6}	2×10^{-6}	1×10^{-6}	4×10^{-6}	4×10^{-7}	1×10^{-6}	1×10^{-6}	3×10^{-6}	1×10^{-6}	NS	***	*	NS	NS	**	NS
Selenomonas ruminantium	0.009	0.011	0.001	0.010	0.009	0.001	0.009	0.011	0.001	t	NS	*	NS	NS	*	NS
Prevotella spp	0.114	0.123	0.008	0.113	0.123	0.011	0.105	0.131	0.011	NS	NS	*	NS	NS	NS	NS
Prevotella ruminicola	0.046	0.053	0.004	0.047	0.051	0.005	0.044	0.055	0.006	NS	NS	NS	***	NS	NS	NS
Prevotella brevis	0.041	0.039	0.006	0.025	0.054	0.007	0.034	0.045	0.008	NS	**	NS	NS	NS	NS	NS
Methanosphaera stadtmanae	0.001	0.001	0.0002	0.001	0.001	0.0002	0.001	0.001	0.0002	NS	NS	*	NS	NS	*	NS
Methanobrevibacter spp.	0.001	0.002	0.0002	0.002	0.001	0.0003	0.001	0.002	0.0003	NS	***	***	NS	NS	***	NS
Methanobacteriales	0.001	0.002	0.0001	0.002	0.001	0.0002	0.001	0.002	0.0002	***	*	***	NS	*	**	NS

* $p < 0.05$, ** $p < 0.01$, *** $p < 0.001$, t = trend $p < 0.10$.[a] SEM: Standard error of the means. 20HF (n = 11 goats): 20g *Schizochytrium spp* and high forage diet (60:40); 20HG (n = 11 goats): 20g *Schizochytrium spp* and high grain diet (40:60); 40HF (n = 11 goats): 40g *Schizochytrium spp* and high forage diet (60:40); 40HG (n = 11 goats): 40g *Schizochytrium spp* and high grain diet (40:60).

Table S6. The mean individual fatty acids (FA) (% of total FA) in rumen fluid of goats fed diets (20HF, 20HG, 40HF, and 40HG) with different levels of microalgae Schizochytrium spp (20 g and 40 g/day/goat) and two different forage to concentrate ratios (60:40 and 40:60) throughout the experimental period (21th and 42th experimental day) investigating the three major factors (forage-to-concentrate ratio, algae level, and sampling time).

	**Dietary treatment (D)**						**Sampling time (S)**			**Effect**						
	Forage/Concentrate			Algae level			Sampling day			Effect			Interaction effect			
	60/40	40/60	SEM[a]	20g	40g	SEM[a]	21	42	SEM[a]	F/C	ALG	TIME	AxF/C	TxF/C	AxT	AxTxF/C
$C_{6:0}$	1.09	1.08	0.164	0.458^{a}	1.72^{b}	0.185	1.10	1.08	0.204	NS	***	NS	NS	**	NS	NS
$C_{8:0}$	1.97	1.60	0.192	1.63	1.95	0.222	1.95	1.63	0.219	NS	t	t	NS	***	**	NS
$C_{10:0}$	1.11	1.12	0.103	0.858^{a}	1.37^{b}	0.126	1.09	1.14	0.144	NS	***	NS	NS	NS	NS	NS
$C_{12:0}$	0.751^{a}	1.02^{b}	0.074	0.681^{a}	1.09^{b}	0.118	0.976	0.791	0.107	*	**	NS	NS	NS	NS	NS
$C_{14:0}$	4.22	4.10	0.159	3.55^{a}	4.77^{b}	0.210	4.45^{a}	3.87^{b}	0.214	NS	***	*	**	NS	NS	**
$C_{14:1}$	1.13	1.03	0.065	1.10	1.06	0.069	1.14^{a}	1.02^{b}	0.071	NS	NS	*	NS	NS	NS	NS
$C_{15:0}$	1.11^{a}	0.909^{b}	0.066	0.991	1.03	0.070	1.03	0.993	0.074	*	NS	NS	NS	NS	NS	NS
$C_{16:0}$	28.51	28.69	0.239	28.21^{a}	28.99^{b}	0.299	28.39	28.81	0.312	NS	**	NS	NS	NS	NS	NS
$C_{16:1\ n\text{-}7}$	0.518	0.581	0.077	0.468^{a}	0.631^{b}	0.089	0.617	0.482	0.088	NS	*	t	NS	NS	NS	NS
$C_{17:0}$	0.871	0.695	0.114	0.570^{a}	0.996^{b}	0.131	0.849	0.717	0.148	NS	***	NS	NS	NS	*	NS
$C_{18:0}$	15.98	11.60	2.264	19.26^{a}	8.33^{b}	2.667	13.68	13.90	2.308	NS	***	NS	NS	NS	**	NS
$C_{18:1\ trans}$	1.86	1.99	0.134	1.89	1.96	0.212	1.84	2.01	0.156	NS	NS	NS	NS	NS	NS	NS
$C_{18:1\ trans\ 11}$	10.97^{a}	18.67^{b}	2.045	12.92	16.71	2.444	14.76	14.88	2.065	*	t	NS	NS	NS	NS	NS
$C_{18:1\ trans\ 10}$	1.18	1.54	0.121	1.33	1.39	0.163	1.34	1.38	0.129	t	NS	NS	NS	NS	NS	NS
$C_{18:1\ cis\text{-}9}$	9.56^{a}	10.78^{b}	0.402	10.20	10.15	0.484	10.18	10.16	0.465	*	NS	NS	NS	NS	*	NS
$C_{18:2\ n\text{-}6\ trans}$	0.745	0.640	0.072	0.795^{a}	0.590^{b}	0.094	0.737	0.648	0.083	NS	*	NS	NS	**	*	**
$C_{18:2\ n\text{-}6\ cis}$	4.14	3.56	0.222	4.69^{a}	3.01^{b}	0.257	3.83	3.87	0.250	t	***	NS	NS	NS	NS	NS
$C_{18:3\ n\text{-}3}$	1.40^{a}	0.887^{b}	0.110	1.03	1.25	0.138	1.16	1.12	0.120	**	t	NS	NS	NS	*	NS
$C_{18:2\ cis\text{-}9,trans\text{-}11}$	1.23^{a}	0.965^{b}	0.080	1.23^{a}	0.971^{b}	0.103	1.00^{a}	1.19^{b}	0.097	*	*	*	NS	NS	NS	NS
$C_{20:3\ n\text{-}3}$	0.022	0.059	0.019	0.071^{a}	0.010^{b}	0.024	0.032	0.049	0.025	NS	*	NS	NS	*	NS	**
$C_{22:5\ n\text{-}6}$	3.41^{a}	2.62^{b}	0.131	2.36^{a}	3.67^{b}	0.185	2.96	3.07	0.161	***	***	NS	*	NS	NS	NS
$C_{22:6\ n\text{-}3}$	8.19^{a}	5.80^{b}	0.322	5.66^{a}	8.33^{b}	0.433	6.88	7.11	0.395	***	***	NS	NS	NS	NS	NS

* $p < 0.05$, ** $p < 0.01$, *** $p < 0.001$, t = trend $p < 0.10$.

[a] SEM: Standard error of the means.

20HF (n = 11 goats): 20g *Schizochytrium spp* and high forage diet (60:40); 20HG (n = 11 goats): 20g *Schizochytrium spp* and high grain diet (40:60); 40HF (n = 11 goats): 40g *Schizochytrium spp* and high forage diet (60:40); 40HG (n = 11 goats): 40g *Schizochytrium spp* and high grain diet (40:60).

Table S7. The mean rumen pH values, ammonia concentration, a-amylase, protease, cellulase, and xylanase activity of goats fed diets (20HF, 20HG, 40HF, and 40HG) with different levels of microalgae *Schizochytrium spp* (20 g and 40 g/day/goat) and two different forage to concentrate ratios (60:40 and 40:60) throughout the experimental period (21th and 42th experimental day) using a GLM model for three-way repeated measures.

	**Dietary treatment (D)**						**Sampling time (S)**			**Effect**						
	Forage/Concentrate			Algae level			Sampling day			Effect			Interaction effect			
	60/40	40/60	SEM[a]	20g	40g	SEM[1]	21	42	SEM[1]	F/C	ALG	TIME	F/C×A	F/C×T	A×T	F/C×A×T
Protease activity (U/ml) [a]	0.043	0.041	0.008	0.038	0.047	0.010	0.053	0.032	0.009	NS	NS	*	NS	NS	NS	NS
a-Amylase activity (U/ml) [b]	2.53	3.12	0.225	3.14	2.51	0.268	2.95	2.70	0.253	t	**	NS	t	NS	NS	NS
Cellulase activity (U/ml/h) [c]	8.70	9.57	0.294	8.76	9.51	0.356	9.20	9.07	0.331	*	*	NS	NS	NS	NS	NS
Xylanase (U/ml/h) [d]	7.73	8.54	0.215	8.08	8.19	0.266	8.22	8.05	0.255	*	NS	NS	NS	NS	NS	NS
NH3-N mg/L	148.3	120.8	9.73	137.6	131.5	10.55	141.5	127.6	10.49	*	NS	*	NS	NS	NS	NS
pH	6.77	6.70	0.101	6.76	6.71	0.125	6.73	6.74	0.099	NS	NS	NS	NS	NS	NS	NS

* $p < 0.05$, ** $p < 0.01$, *** $p < 0.001$, t = trend $p < 0.10$.
[1] SEM: Standard error of the means.

[a] Units/ml= [(μmole of protein equivalent to ABS) x (total volume of reaction in ml) x dilution factor] / [(incubation time in minutes) x (volume of colorimetric determination in ml) x (volume of rumen sample in ml)]
[b] One unit of α-amylase activity will liberate 1.0 mg of maltose from starch in 10 min at pH 6.9 at 39°C
[c] One unit of cellulase activity based on endo-cellulase (*A. niger*) clearance activity in 24h incubation at 50°C.
[d] One unit of xylanase activity based on endo-1-4-beta-Xylanase M1 (*T. viride*) clearance activity in 24h incubation at 37°C.

Table S8. The mean rumen pH values, ammonia concentration, a-amylase, protease, cellulase, and xylanase activity of goats fed diets (20HF, 20HG, 40HF, and 40HG) with different levels of microalgae *Schizochytrium spp* (20 g and 40 g/day/goat) and two different forage to concentrate ratios (60:40 and 40:60) throughout the experimental period (21th and 42th experimental day).

	**Dietary treatment (D)**					**Sampling time (S)**			**Effect**		
	20HF	20HG	40HF	40HG	SEM[1]	21	42	SEM[1]	D	T	D x T
Protease activity (U/ml) [a]	0.039	0.036	0.040	0.046	0.010	0.049	0.031	0.007	NS	t	NS
a-Amylase activity (U/ml) [b]	2.62^{b}	3.65^{a}	2.41^{b}	2.58^{b}	0.263	2.94	2.69	0.151	**	NS	NS
Cellulase activity (U/ml/h) [c]	8.09^{a}	9.44^{b}	8.86^{ab}	9.71^{b}	0.330	9.02	9.02	0.198	**	NS	NS
Xylanase (U/ml/h) [d]	7.55^{a}	8.62^{b}	7.84^{a}	8.47^{b}	0.266	8.21	8.03	0.152	*	NS	NS
NH3-N mg/L	155.95^{a}	119.29^{b}	140.73^{ab}	122.26^{b}	10.366	141.19^{a}	127.93^{b}	5.993	*	*	NS
pH	6.75	6.77	6.79	6.63	0.125	6.75	6.69	0.099	NS	NS	NS

Means with different superscript letters (a, b) between dietary groups and sampling time differ significantly; * $p < 0.05$, ** $p < 0.01$, *** $p < 0.001$, t = trend $p < 0.10$.
[1] SEM: Standard error of the means.

[a] Units/ml= [(μmole of protein equivalent to ABS) x (total volume of reaction in ml) x dilution factor] / [(incubation time in minutes) x (volume of colorimetric determination in ml) x (volume of rumen sample in ml)]
[b] One unit of α-amylase activity will liberate 1.0 mg of maltose from starch in 10 min at pH 6.9 at 39°C
[c] One unit of cellulase activity based on endo-cellulase (*A. niger*) clearance activity in 24h incubation at 50°C.
[d] One unit of xylanase activity based on endo-1-4-beta-Xylanase M1 (*T. viride*) clearance activity in 24h incubation at 37°C.
